# Full high-throughput sequencing analysis of differences in expression profiles of long noncoding RNAs and their mechanisms of action in systemic lupus erythematosus

**DOI:** 10.1186/s13075-019-1853-7

**Published:** 2019-03-05

**Authors:** Hui Ye, Xue Wang, Lei Wang, Xiaoying Chu, Xuanxuan Hu, Li Sun, Minghua Jiang, Hong Wang, Zihan Wang, Han Zhao, Xinyu Yang, Jianguang Wang

**Affiliations:** 1 Department of Biochemistry, School of Basic Medical Sciences, Wenzhou Medical University, Wenzhou, 325035 Zhejiang China; 2 School of the Second Clinical Medical Sciences, Wenzhou Medical University, Wenzhou, 325035 Zhejiang China; 3 The First Affiliated Hospital of Wenzhou Medical University, Wenzhou, 325035 Zhejiang China; 4 The Second Affiliated Hospital of Wenzhou Medical University, Wenzhou, 325035 Zhejiang China; 5 School of Stomatology, Wenzhou Medical University, Wenzhou, 325035 Zhejiang China; 6 Department of Medicinal Chemistry, School of Pharmaceutical Sciences, Wenzhou Medical University, Wenzhou, 325035 Zhejiang China

**Keywords:** High-throughput sequencing analysis, Expression profiles, Long noncoding RNA, Mechanisms, Systemic lupus erythematosus

## Abstract

**Background:**

The specific function of long noncoding RNAs (lncRNAs) in systemic lupus erythematosus (SLE) and the mechanism of their involvement in related pathological changes remain to be elucidated, so, in this study, we analyzed the differences in the expression profiles of lncRNAs and their mechanisms of action in SLE using full high-throughput sequencing, bioinformatics, etc. methods.

**Methods:**

We used high-throughput sequencing to detect differences in the expression profiles of lncRNAs, miRNAs, and mRNAs in PBMCs from patients with SLE at the genome-wide level. Next, we predicted target genes of 30 lincRNAs (long intergenic noncoding RNAs) by constructing a coexpression network of differential lincRNAs and mRNAs and identified the role of lincRNAs. Then, we analyzed the coexpression network of 23 optimized lincRNAs and their corresponding 353 miRNAs, evaluated the *cis*- and *trans*-effects of these lincRNAs, and performed GO and KEGG analyses of target genes. We also selected 8 lincRNAs and 2 newly discovered lncRNAs for q-PCR validation and lncRNA–miRNA–mRNA analysis. Finally, we also analyzed respectively the relation between lncRNAs and gender bias in SLE patients using RT-qPCR, the relation between Systemic Lupus Erythematosus Disease Activity Index score and the “IFN signature” using ELISA, and the relation between the differential expression of lncRNAs and a change in the number of a cell type of PBMCs in SLE patients using RT-qPCR.

**Results:**

The profiles of 1087 lncRNAs, 102 miRNAs, and 4101 mRNAs in PBMCs significantly differed between patients with SLE and healthy controls. The coexpression network analysis showed that the network contained 23 lincRNAs and 353 mRNAs. The evaluation of the *cis*- and *trans*-effects showed that the 23 lincRNAs acted on 704 target genes. GO and KEGG analyses of the target genes predicted the biological functions of the 23 lincRNAs. q-PCR validation showed 7 lincRNAs and 2 novel lncRNAs were identical to the sequencing results. The ceRNA network contained 7 validated lincRNAs, 15 miRNAs, and 155 mRNAs. In addition, the differential expression of lncRNAs may be gender dependent in SLE patients, SLE patients also exhibit a robust “IFN signature,” and PBMCs exhibiting differential expression of lncRNAs may be due to a change in the number of a cell type.

**Conclusion:**

This work determined specific lncRNAs that play important biological functions in the pathogenesis of lupus and provided a new direction for diagnosis and treatment of disease.

**Electronic supplementary material:**

The online version of this article (10.1186/s13075-019-1853-7) contains supplementary material, which is available to authorized users.

## Introduction

Systemic lupus erythematosus (SLE) is a chronic autoimmune disease with complicated clinical manifestations and leads to multiple systemic impairments. The course of the disease repeats, and aggravation and remission alternate; however, the early symptoms are often atypical [1]. The main features of SLE are the presence of numerous pathogenic autoantibodies, complement depletion, and chronic inflammation. SLE has a clear gender tendency; that is, it mainly affects women of childbearing age (a ratio of 1:9 for men to women). According to statistics, 150 cases of SLE occur among 100,000 people. Among people suffering from SLE for 5 years, those with kidney failure or died account for 15% [2]. The development of SLE is jointly influenced by various factors, such as genes, environment, epigenetics, and hormones. However, the precise pathogenesis of SLE remains unclear.

Long noncoding RNAs (lncRNAs) are endogenous transcript RNA molecules that have a length of more than 200 nucleotides [3] and have no protein-encoding capacity. LncRNAs were previously ignored and considered as nonfunctional mRNA transcription by-product. LncRNA was first discovered in 2002 during a large-scale, genome-wide sequencing of the rat cDNA library [4]. Since then, many studies have explored lncRNAs and reported their involvement in regulating the expression of protein-coding genes and epigenetic genes through various mechanisms, such as epigenetic modification, alternative splicing, and post-transcriptional and translational control [5]. LncRNAs are categorized into five groups [3]: (1) long intergenic noncoding RNAs (lincRNA) between genes, (2) antisense lncRNAs, (3) intronic transcript lncRNAs, (4) promoter-associated lncRNAs, and (5) UTR-associated lncRNAs. In the present work, the most studied and functional lincRNAs were analyzed. About 20% of lincRNAs in cells influenced gene expression through epigenetic effects by binding to and targeting chromatin-modifying complexes, such as PRC2 [6]. LncRNAs are thought to be associated with cell differentiation and activation and play an important regulatory role in the differentiation and activation of immune cells in congenital and acquired immune systems [7]. Therefore, lncRNAs could be related to various autoimmune diseases, such as rheumatoid arthritis, psoriasis, and sicca syndrome [8]. Scholars in the field of transcriptome research have focused on determining lncRNAs that are associated with the pathogenesis of SLE. In this disease, differences in the expression of lncRNAs were found. Wu et al. reported the significant downregulation of linc0949 and linc0597 in peripheral blood mononuclear cells (PBMCs) of patients with SLE; the results also indicated that linc0949 expression was negatively correlated with the degree of disease activity and organ damage but positively correlated with complement C3 level [9]. However, the specific mechanism of action of lncRNAs in the development of SLE has not been elucidated yet. Current research on lncRNAs in SLE has relied on the results of ChIP analysis [9, 10] and did not perform high-throughput sequencing.

Comprehensively understanding the epigenetic and molecular mechanisms of SLE is the key to achieve early diagnosis, recommend appropriate treatment, and obtain good prognosis. This study uses full high-throughput sequencing technology to analyze the differences in lncRNA expression profiles between patients with SLE and controls (healthy individuals) at the whole-genome level. Specific lincRNA target genes were predicted, and lncRNA–miRNA–mRNA ceRNA networks were constructed. In addition, we also make experiments to confirm the following: whether differential expression of lncRNAs in our screen was gender dependent, whether SLE patients exhibited a robust or weak “IFN signature,” and whether PBMCs from SLE patients (as compared with healthy donors) exhibited differential expression of lncRNAs due to a change in the number of a cell type (e.g., lymphocytes, monocytes, and DCs) in peripheral blood. Results would be used as the basis for the study of specific lncRNAs involved in the specific pathogenesis of SLE.

## Methods

### Participants and sample collection

This study recruited 147 individuals with SLE (aged 41.3 ± 10.6 years) who were outpatients or inpatients of the Rheumatology and Immunology Division of the First Affiliated Hospital of Wenzhou Medical University and the Second Affiliated Hospital of Wenzhou Medical University from March 2016 to October 2018. The selected cases were consistent with the SLE diagnostic criteria revised by the American Rheumatism Association in 1997. Blood specimen collection and Systemic Lupus Erythematosus Disease Activity Index (SLEDAI) [11] statistical analysis were conducted before administering glucocorticoids and immunosuppressive agents to the patients. The normal control group consisted of 117 healthy donors (aged 40.8 ± 12.1 years) with no history of autoimmune diseases or immunosuppressive therapy. The control group matched the patient group in terms of age and gender. This study was approved by the Research Ethics Committee of Wenzhou Medical University (No. 2018925) and obtained informed consents from all participants. In high-throughput sequencing, 30 patients with SLE and 30 healthy donors were divided into 10 groups (among them, patients were grouped according to their SLEDAI scores: group 1 consisted of individuals with no activity (3.2 ± 0.9 points), group 2 (6.3 ± 0.7 points) and group 3 (8.3 ± 0.5 points) consisted of those with mild activity, group 4 consisted of those with moderate activity (13.0 ± 1.2 points), and group 5 consisted of those with severe activity (19.0 ± 2.6 points); each group was mixed and sequenced with 6 samples of the same SLEDAI score). In the study of a relation between lncRNAs and gender bias in SLE patients, 37 healthy donors, 19 SLE patients (containing male and female), 26 female SLE patients, and 13 male SLE patients were used for RT-qPCR. In the study of a relation between SLEDAI score and the “IFN signature,” 33 SLE patients (12 inactive (SLEDAI score < 4) and 21 active (SLEDAI score > 4)) and 20 healthy donors were used for enzyme-linked immunosorbent assay (ELISA). In the study of a relation between the differential expression of lncRNAs and a change in the number of a cell type of PBMCs in SLE patients, 26 SLE patients and 30 healthy donors were used for the isolation of PBMCs, T cells, B cells, monocytes, DCs, and RT-qPCR.

### High-throughput sequencing analysis

High-throughput sequencing was completed by RiboBio Co. Ltd. (Guangzhou, China). The operation procedure was briefly described as follows: After total RNA extraction, the Ribo-ZeroTM rRNA Removal Kit (Epicenter, Madison, WI, USA) was used to remove rRNA and achieve RNA fragmentation (average fragment length of approximately 200 nt). Single-stranded cDNA was synthesized by reverse transcription, and then double-stranded cDNA was synthesized. After purification of double-stranded cDNA, the terminal repair was performed. The sample was added with primers, amplified, and purified through PCR for library construction according to RNA species. After the quality inspection of the library, ten sets of samples (five vs. five) were sequenced using the Illumina HiSeqTM 2500 sequencing platform (Illumina, San Diego, CA, USA). Bioinformatics analysis of raw sequence data was conducted, and the ensemble transcript database was used to annotate the results.

### Real-time quantitative reverse transcriptase polymerase chain reaction validation

The expressions of selected lncRNAs were screened and verified by qRT-PCR. Glyceraldehyde 3-phosphate dehydrogenase (GAPDH) mRNA was used as an internal control. The primers are listed in Additional file 1: Table S1. For reverse transcription (RT) reactions, SuperScript First-Strand Synthesis System (Invitrogen, Carlsbad, CA, USA) was used. In brief, random primers were used and reacted at 42 °C for 60 min and at 72 °C for 10 min to obtain a cDNA template. SYBR Green PCR Master Mix (Applied Biosystems, Foster City, CA, USA) was used for PCR amplification. The reaction system was added with 10 μl 2× SYBR Green mix, 6.8 μl of cDNA, 0.8 μl upstream primer (5 μM), 0.8 μl downstream primer (5 μM), and 1.6 μl of RNase-free H_2_O up to the total volume of 20 μl. All experiments were performed in triplicates. The final qPCR data of lncRNA were processed using 2ΔΔct method against GAPDH for normalization.

### Prediction of 30 screened lincRNA target gene

The function of 30 screened lincRNA was predicted through bioinformatics analysis to determine its effect on target genes. Previous works indicated that the lincRNA gene could regulate post-transcriptional steps. The role of lincRNAs and their target genes was predicted by establishing the relationship between differential lincRNAs and differential mRNAs. The roles of lincRNAs and their target genes included *cis*- and *trans*-actions [12]. The lincRNA *cis*-action is predicted by searching all coding genes within the 10-kb upstream and downstream of the target lincRNA, and these neighboring genes may be regulated by lincRNAs. The lincRNA *trans*-action is predicted based on nucleic acid base pairing.

### Gene Ontology and Kyoto Encyclopedia of Genes and Genomes analysis

Gene Ontology (GO) analysis annotates differentially expressed genes in terms of cell composition, molecular function, and biological processes [13]. Kyoto Encyclopedia of Genes and Genomes (KEGG) analysis is an effective method for predicting the potential biological function of differentially expressed genes [14]. KEGG analysis is also used to provide differentially expressed mRNAs with annotation information for signal transduction and disease pathways and background knowledge of gene pathways and functional studies. In the GO and KEGG analyses, *P* value < 0.05 was used as the screening standard.

### The correlation between IFN-α and disease activity in SLE patients

The venous blood of 33 SLE patients (12 in the no-activity group (SLEDAI score < 4) and 21 in the activity group (SLEDAI score > 4)) and 20 healthy donors (see the “Participants and sample collection” section for details) was collected aseptically (5 ml per person) and left undisturbed for 2 h at room temperature. After serum separation, it was centrifuged (500*g* × 10 min) to aspirate the supernatant serum, which was dispensed into an Eppendorf tube and stored in a refrigerator at − 20 °C. According to the manufacturer’s instructions, an interferon (IFN)-α ELISA Kit (purchased from PBL Biomedical Laboratories, USA) was used to detect the IFN-α level in SLE patients and normal control group. The operation procedure was briefly described as follows: The diluted coated antibody was added to 100 μl pores of ELISA plate and stored for 48 h at 4 °C. The ELISA plate was washed three times with washing solution, and then 200 μl of blocking buffer per well was added for 1 h at 37 °C. the ELISA plate was washed three times, and the sample to be tested was added with the negative control; the double-diluted enzyme-labeled antibodies were added, 100 μl per well; they were placed at 37 °C for 1 h; the ELISA plate was washed three times; the prepared ABTS color-substrate solution was added, 100 μl per well; at 37 °C, the color was developed for 30 min by water bath; the optical density (OD) values at 410 nm were measured with an ELISA reader; a standard curve was drawn; the corresponding results of each serum sample were determined, and then Prism Software was used to make a scatter plot and a correlation analysis graph.

### Isolation of PBMCs, T cells, B cells, monocytes, DCs, and RT-qPCR of lncRNA NR_034053.2

The whole blood of 26 SLE patients and 30 healthy donors (see the “Participants and sample collection” section for details) was collected aseptically (30 ml per person) in EDTA collection tubes, and PBMCs were isolated by density-gradient centrifugation with Ficoll-Paque Premium (GE Healthcate), according to the instructions. For the subsets of PBMCs isolation, the fresh PBMCs were incubated for 15 min at 4 °C with fluorescent-conjugated monoclonal antibodies: anti-CD3-PerCP-Cy5.5, antiCD14-PE, and anti-CD19-APC (all from BD Biosciences). Stained cells were sorted on a BD FACSAria III (BD Biosciences). T cells were identified as CD3^+^/CD19^−^. B cells were collected if cells were CD19^+^/CD3^−^. Monocytes were isolated if cells were CD14^+^/CD3^−^. And then, monocyte cells were then cultured for 5–7 days in RPMI 1640 supplemented with 1000 U/ml granulocyte/macrophage colony-stimulating factor (GM-CSF) and 1000 U/ml interleukin (IL)-4 (PeproTech, Rocky Hill). For monocyte-derived dendritic cell (moDC) maturation, 1 μg/ml lipopolysaccharide (LPS; *Escherichia coli* type 055:B6; Sigma) was added to the medium at day 6.

Then, the total RNAs of PBMC, T cells, monocytes, B cells, and DCs of the above SLE patients were extracted respectively, and the total RNAs from PBMC and DCs of healthy donors were extracted; next, RT-qPCR for lncRNA NR_034053.2 of the above cells was performed respectively, and internal reference was GAPDH (note: for the primer sequence, please see Additional file 1: Table S1).

### Construction of LncRNA–mRNA coexpression network

An lncRNA–mRNA coexpression network was constructed based on the specific expression levels of mRNAs and the normalized signal intensity of lncRNA to determine the interaction between differentially expressed mRNAs and differentially expressed lncRNAs. After obtaining the expression values of all genes, their correlation with one another and *P* value were calculated and screened (COR > 0.85). A given node would be filtered out if the Pearson correlation coefficient between any pair of nodes is greater than COR. Only COR value was used for screening.

### LncRNA–miRNA–mRNA network analysis

The expression profiles of differentially expressed lncRNAs, miRNAs, and mRNAs and the prediction feature of bioinformatics software were used to construct lncRNA–miRNA–mRNA networks. miRanda, PITA, and RNAhybrid were used to predict the recognition regions of lncRNAs and miRNAs. Further screening was conducted, and the prediction results of each software program were collated. Two or more software predictions were used as candidate reciprocal miRNAs for lncRNAs. For candidate miRNAs, miRDB, miRTarBase, miRWalk, and TargetScan software were used. The results of the three software programs and the common prediction results were regarded as miRNAs corresponding to mRNAs. The lncRNAs and their candidate miRNAs and corresponding mRNAs were selected.

### Statistical analysis

Continuous variables are expressed as means (SD). The Student *t* test or one-way analysis of variance was used to compare continuous variables. All *P* values were estimated in a two-tailed fashion. Differences were considered to be statistically significant at *P* < 0.05. Data were analyzed using SPSS 22.0 (SPSS Inc., IL, USA). The relationships between the expression levels of IFN-α and Systemic Lupus Erythematosus Disease Activity Index (SLEDAI) were analyzed by Pearson’s correlation coefficient. Mapping was completed using GraphPad Prism 6. To highlight the differences in the expression during sequencing, we used Audics for correcting the obtained *P* value to the *q* value; the lower the *q* value is, the more significant the difference in gene expression will be.

## Results

### Expression profiles of differentially expressed lncRNAs, miRNAs, and mRNAs

**Fig. 1:**
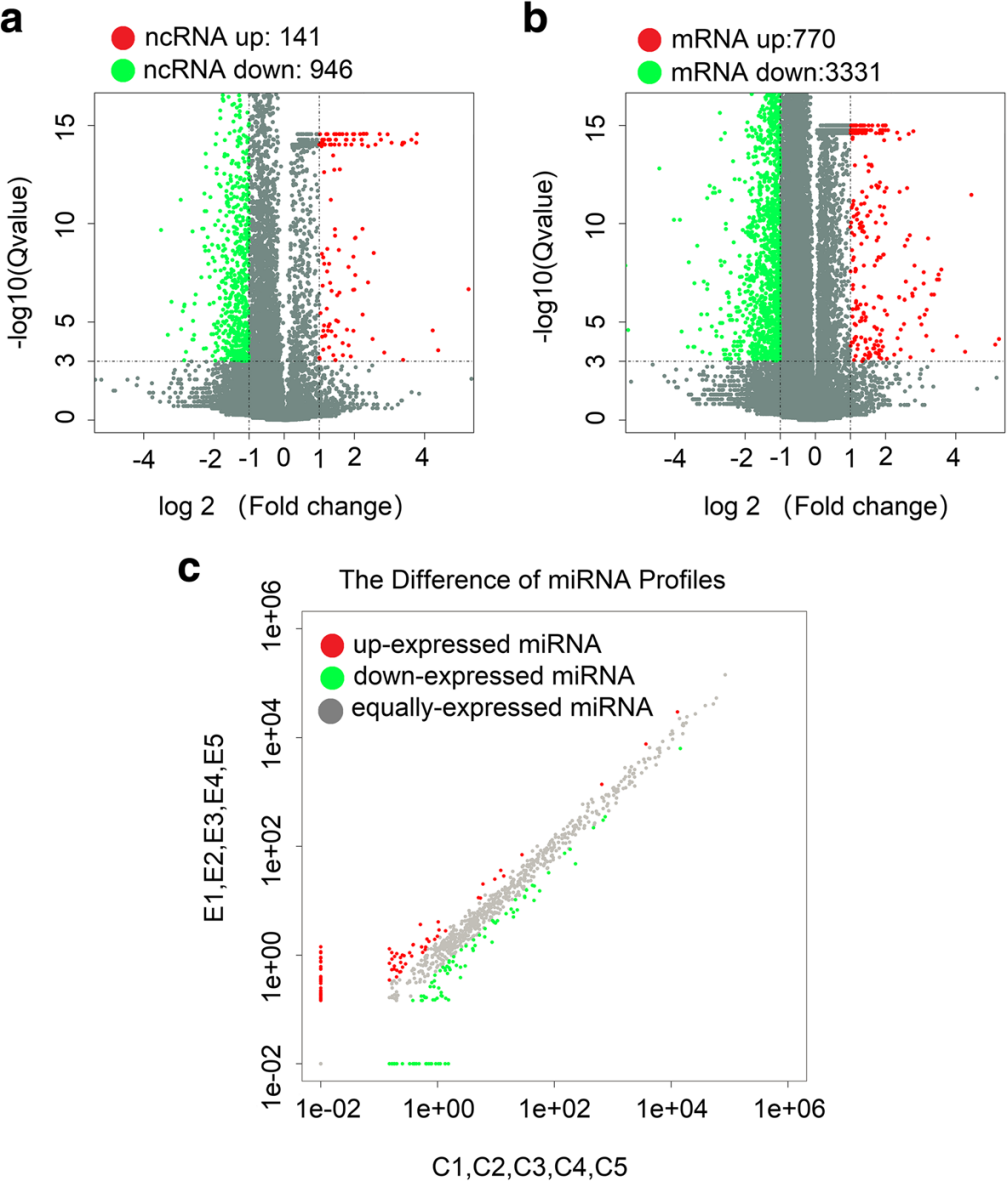
High-throughput sequencing to reveal differences in the expression profiles of lncRNAs, mRNAs, and miRNAs between the SLE and control groups. **a** Differentially expressed lncRNA volcano maps between the SLE and NC groups. **b** Differentially expressed mRNA volcano plots between the SLE and NC groups. **c** Differentially expressed miRNA scatter maps between the SLE and NC groups

Second-generation sequencing technology was used to detect lncRNAs in PBMCs in the five groups of patients with SLE and the five groups of healthy controls. The application of volcano plots was used to directly show the overall distribution of lncRNAs and mRNAs in the two groups, and scatter maps were used to demonstrate the distribution of miRNAs (Fig. 1a–c). Using ∣log2(fold_change)∣1, *q* < 0.001 as the standard, we determined significant expression differences in 1087 lncRNAs (141 significant upregulations and 946 significant downregulations). Among the 47 lncRNAs that differed more than fivefold in number, 25 were upregulated and 22 were downregulated. ENST00000437947.1 upregulation is the most obvious (fold change up, 38.11160073), and NR_006881.1 downregulation is the most obvious (fold change down, 66.77770783). Of these differentially expressed lncRNAs, 184 belong to lincRNAs (33 were significantly upregulated and 151 were significantly downregulated), and 220 belong to antisense lncRNAs (37 were significantly upregulated and 183 were significantly downregulated).

**Fig. 2:**
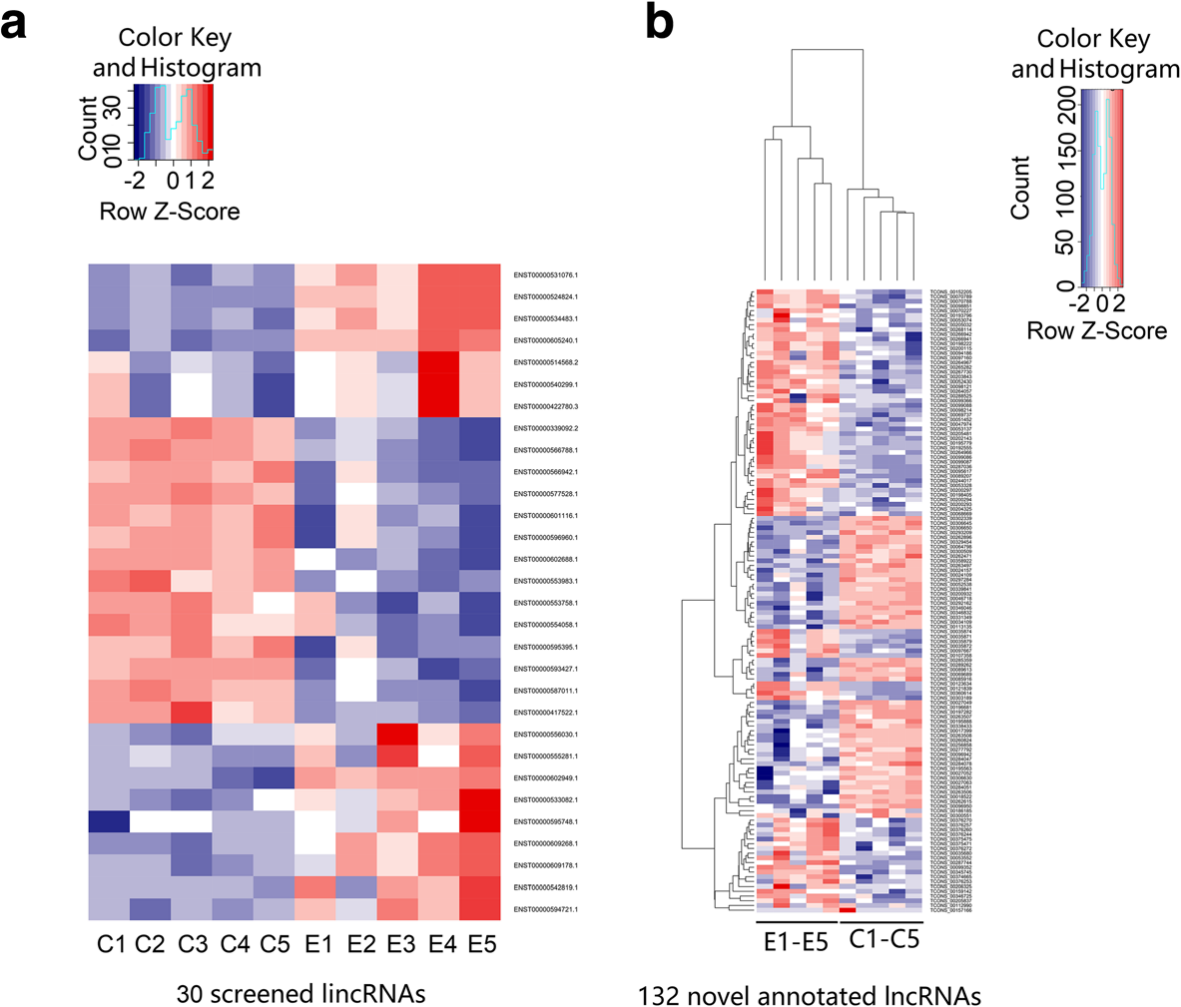
Cluster heat map analysis. **a** Thirty screened lincRNAs. **b** One hundred thirty-two novel annotated lncRNAs

In recent years, the role of lincRNAs has been described in several diseases and has become another star molecule following microRNA. After comprehensive analysis of the expression volume (RPKM), differential multiples, and lincRNA-derived genes, we screened 30 lincRNAs (15 upregulated and 15 downregulated) located near the SLE susceptibility locus for further study. The selected lincRNAs are shown in Additional file 2: Table S2 and Additional file 3: Table S3. We used a clustering heat map (Fig. 2a) to show the differences in the expression levels of the 30 lincRNAs between SLE and the control groups. We found 102 differential miRNAs (39 significantly upregulated and 63 significantly downregulated) and 4101 differential mRNAs (770 significantly upregulated and 3331 significantly downregulated). The discovery of these transcript differences laid the foundation for exploring the pathogenesis of lupus at the molecular level.

### Identification and differential expression analysis of novel lncRNAs

A large number of novel promising lncRNAs in SLE have yet to be further screened because of the limitation of using microarray technology, which could only focus on known candidate lncRNAs preset on the chip. High-throughput sequencing not only can detect the expression of known lncRNAs but also can discover novel lncRNAs. In this study, we identified novel lncRNAs through the following stringent filter steps: (1) filter genes and lncRNAs with known filter database, (2) identify RNAs longer than 200 nt, (3) determine RNAs with predicted open reading frames (ORF) < 300 nt, (4) filter RNA with protein domain (Pfam), and (5) filter RNA with coding potential. We screened transcripts with lncRNA characteristics in new transcripts step-by-step and defined a novel lncRNA with a coding potential score of less than − 1 in Coding Potential Calculator (CPC) software. We found a total of 5029 novel lncRNAs; among which, 132 showed significant differences and included 77 with significantly high expression and 55 with significantly low expression (∣log2(fold_change)∣ > 1, *q* < 0.001). A clustering heat map was drawn to show the significant differences (Fig. 2b). Fifty novel lncRNAs had a difference multiple of 5 times and included 26 upregulated and 24 downregulated. The expression of TCONS_00205837 increased the most (fold change up, 67.72275292), whereas that of TCONS_00157166 decreased the most (fold change down, 51.89499278). We selected novel lncRNAs located near the SLE susceptibility locus for q-PCR validation. TCONS_00195779 was located at 2p25.2, where Linc00487 was also located. Linc00487 was confirmed to be related to B cell development. TCONS_00027049 and IL-10 were located at 1q31.3. IL-10 was found to be involved in immunoregulatory disorders of SLE [15]. Compared with the annotated lncRNAs, novel lncRNAs exhibited higher expression level and more significant differences in the sequencing results. Hence, these novel lncRNAs must be further investigated.

### lincRNAs and two novel lncRNAs showed expression profiles consistent with the sequencing results

**Fig. 3:**
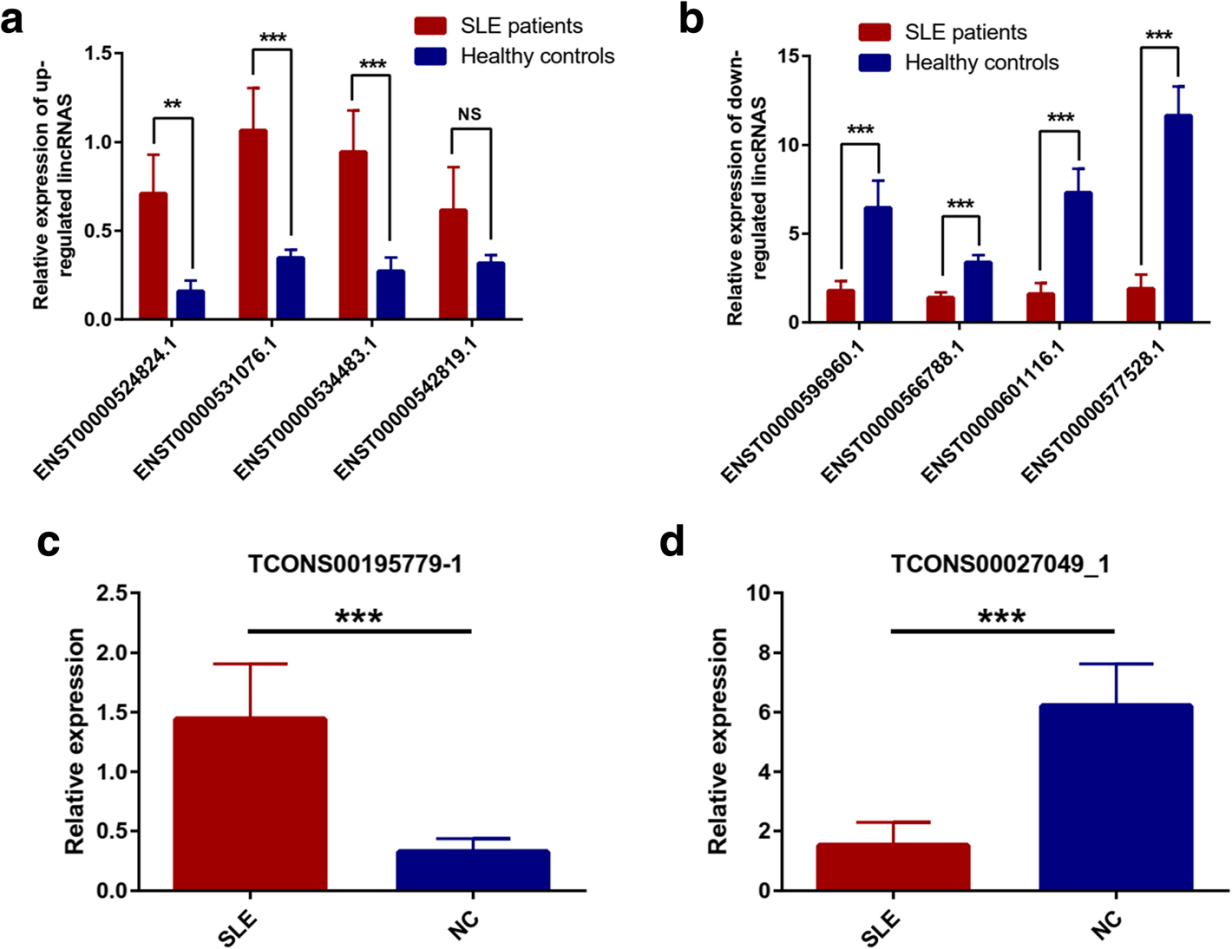
The qRT-PCR validation of 8 selected lincRNAs and 2 novel lncRNAs between 30 SLE patients and healthy controls. **a** Relative expression of 4 upregulated lincRNAs. **b** Relative expression of 4 downregulated lincRNAs. **c** The relative expression of upregulated novel lncRNA TCONS00195779_1. **d** The relative expression of upregulated novel lncRNA TCONS00027049_1

To screen lncRNAs with specific functions and verify the sequencing results, we specifically screened 8 lincRNAs (4 upregulated: ENST00000524824.1, ENST00000531076.1, ENST00000534483.1, and ENST00000542819.1; 4 downregulated: ENST00000596960.1, ENST00000566788.1, ENST00000601116.1, and ENST00000577528.1) and 2 novel lncRNAs (1 upregulated: TCONS00195779_1; 1 downregulated: TCONS00027049_1) for qRT-PCR validation. Samples were obtained from PBMCs of 30 patients with SLE and 30 healthy controls. These lncRNAs have relatively high expression levels, with large difference multiples, and are all located near the susceptibility genes. In the 4 upregulated lincRNAs, 3 lincRNAs were verified successfully, and their difference from ENST00000542819.1 was not statistically significant (Fig. 3a). The ENST00000524824.1 expression increased with the largest multiples (up to 4.49 times). The expression level of ENST00000531076.1 increased 3.07 times and that of ENST00000534483.1 increased 3.47 times. All 4 downregulated lincRNAs were verified successfully. The ENST00000577528.1 expression decreased the most significantly (up to 6.14 times), followed by ENST00000601116.1 (4.59 times) and ENST00000596960.1 (3.61 times), and finally ENST00000566788.1 (2.42 times, Fig. 3b). In addition, differences in the expression of newly discovered lncRNAs were verified by q-PCR (Fig. 3c, d). The expression of TCONS00195779_1 increased 4.39 times and that of TCONS00027049_1 decreased 4.06 times. Seven lincRNAs and 2 novel lncRNAs showed expression profiles consistent with the sequencing results. This finding provides a basis for further analysis of the specific mechanism of action of lncRNAs.

### lincRNAs may act on 704 target genes, forming a total of 890 lncRNA–gene connections

Many studies reported that lncRNAs can act by regulating the transcription of nearby coding genes [16, 17]. LncRNAs can also regulate the expression of genes at the post-transcriptional level by forming double-stranded complexes with mRNA [18]. In the present study, we established the relationship between differential lincRNAs and differential mRNA by predicting lincRNA action mode (*cis*- or *trans*-action). We identified 30 target genes that may act as lincRNAs. The prediction results indicate that 23 of 30 lincRNAs may act on 704 target genes, forming a total of 890 lincRNA–gene connections (Additional file 4: Figure S1). The seven remaining lincRNAs had no potential target genes; of which, ENST00000601116.1 has the most target genes (270); ENST00000534483.1, ENST00000524824.1, and 1ENST00000531076.1, which are from ENSG00000255328.1, have 134 target genes. ENST00000566788.1 from ENSG00000260539.1 also has 134 target genes. ENST00000577528.1 may have effects on 60 target genes.

### GO and pathway analyses of 23 selected lincRNAs on 740 differential target genes

**Fig. 4:**
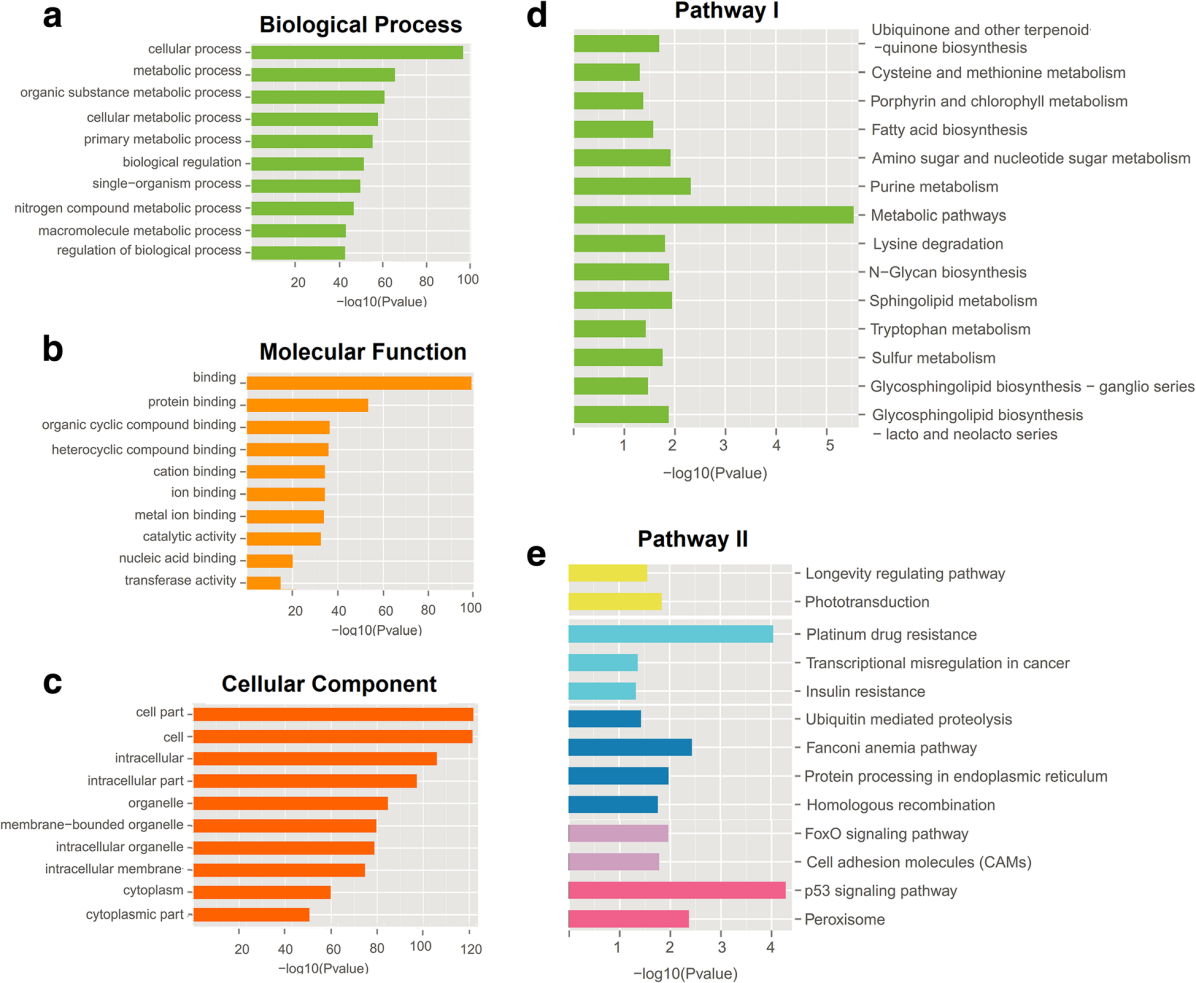
GO and KEGG analyses of target genes. **a**–**c** The highest enrichment GO project among the 30 major entities. **d**, **e** KEGG pathway analyses of target genes: 27 pathways with significant functional changes (including 6 parts of cellular processes, namely, environmental information processing, genetic information processing, human diseases, metabolism, and organismal systems)

To further clarify the biological roles of the 23 selected lincRNAs in SLE, we performed GO and pathway analyses on 740 differential target genes (Fig. 4). The analysis of the KEGG signaling pathway showed that the functional changes in 27 pathways had statistical significant difference between the SLE and the control groups (*P* < 0.05). These pathways included 118 abnormally expressed genes. The pathogenesis of lupus was found to be reacted to the following: metabolic pathways (the highest enrichment, *P* = 2 × 10^−6^), p53 signaling pathway (second highest enrichment, *P* = 5.25 × 10^−5^), and other pathways involved in FoxO signaling (*P* = 0.01), sphingolipid metabolism (*P* = 0.011), and cell adhesion molecules (CAMs) (*P* = 0.016) [19–21]. The GO project enrichment of the target genes included 1175 biological processes, 235 molecular functions, and 207 cellular components (*P* < 0.05), which mainly involves metabolism, cell signal transduction, DNAI binding and transcription, cell cycle, cytoskeleton, cell adhesion, apoptosis, and antigen presentation. The enrichment of cellular processes (*P* = 1.62 × 10^−97^), metabolic processes (*P* = 2.24 × 10^−66^), binding (*P* = 1.58 × 10^−99^), and other GO stems was found to be the highest. Hence, lincRNAs can participate in the pathogenesis of lupus by affecting these functions.

### The differential expression of lncRNAs may be gender dependent in SLE patients

**Fig. 5:**
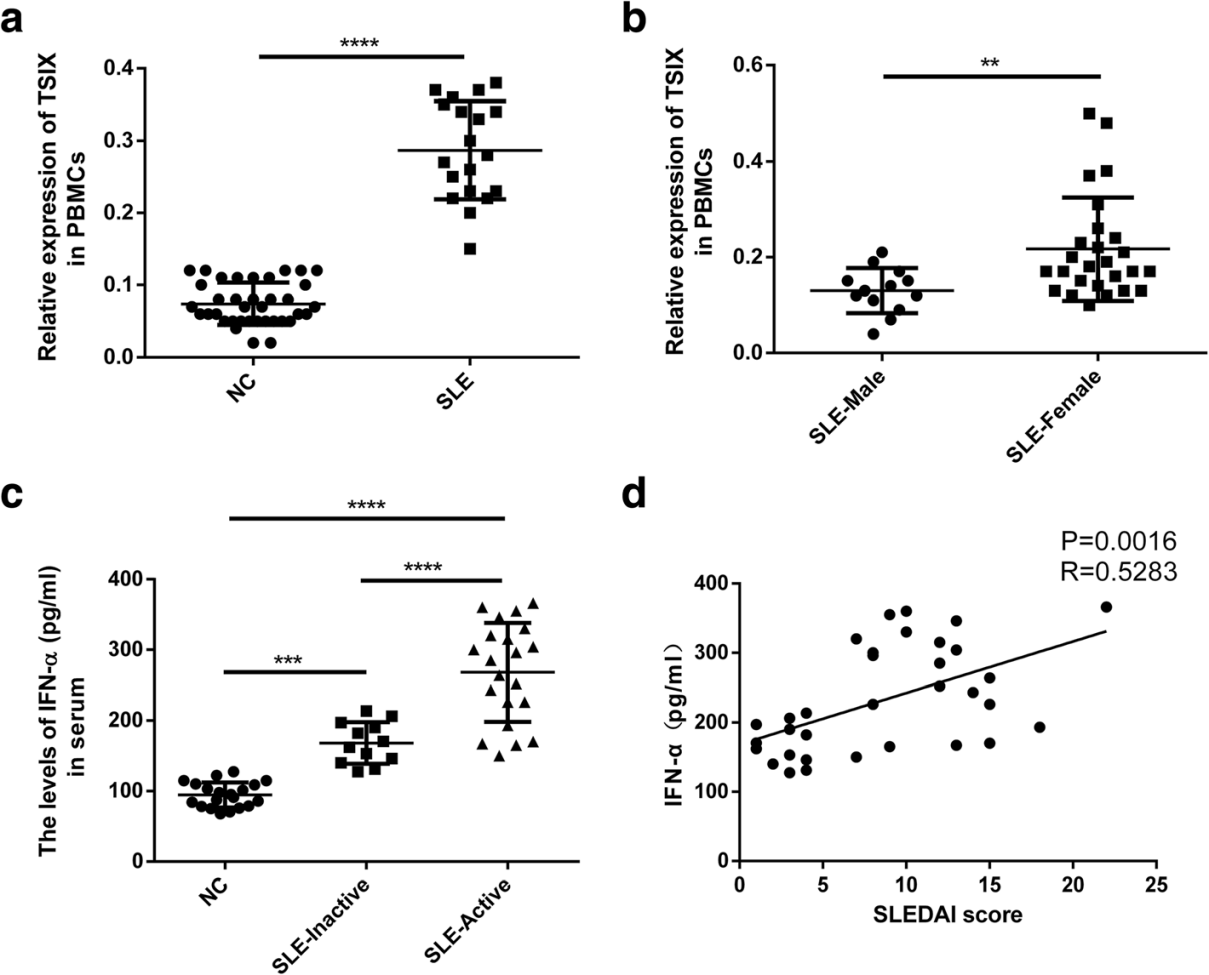
SLE patients exhibit a strong gender bias and a robust “IFN signature.” **a** TSIX expressions in PBMCs of 19 patients with SLE and 37 healthy donors (NC) were analyzed by qPCR. **b** TSIX expressions in PBMCs of 13 male SLE patients and 26 female SLE patients were analyzed by qPCR. **P < 0.01, ***P < 0.001, ****P < 0.0001. **c** IFN-α expressions in the serum of 12 patients with inactive SLE, 21 patients with active SLE, and 20 healthy donors (NC) were analyzed by ELISA. ***P < 0.001, ****P < 0.0001. **d** Nonparametric correlation (Spearman) was performed to assess the correlation between IFN-α and SLEDAI score in patients with SLE (*n* = 33)

SLE in patients exhibits a strong gender bias, so we wondered whether differential expression of lncRNAs in our screen was gender dependent. There is an increased incidence and prevalence of systemic lupus erythematosus in females, which might involve X chromosome inactivation [22]. TSIX is a long noncoding RNA, which protects the active-X from ectopic silencing once X-inactivation has commenced [23]. As shown in Fig. 5a, the expression level of TSIX in SLE patients was significantly higher than that in the Nc group (healthy donors), and the difference was significant (*P* < 0.0001). The expression level of TSIX in female SLE patients was also significantly higher than that in male SLE patients (*P* = 0.003) (Fig. 5b). These results indicated that in SLE patients, the upregulation of TSIX may promote the inactivation of the X chromosome by protecting active-X from ectopic silence.

### A positive correlation between IFN-α and disease activity in SLE patients

Because interferon (IFN)-α is an important regulator of the body’s inflammation and immune response, we wondered whether SLE patients exhibited a robust or weak “IFN signature” and further whether a particular SLEDAI score associated with the “IFN signature” or not. As shown in Fig. 5c, the IFN-α level in the serum of SLE patients is significantly higher than that in the normal control group (healthy donors) with significant difference (*P* < 0.001). The IFN-α level in the serum of SLE patients in the active group is also significantly higher than that in the non-active group (*P* < 0.0001). And there was a positive correlation between IFN-α expression and SLEDAI score in patients with SLE (*R* = 0.5283, *P* = 0.0016) (Fig. 5d). These results indicate that in SLE patients, the overexpressed IFN-α level correlates positively with the disease activity.

### PBMCs exhibiting differential expression of lncRNAs may be due to a change in the number of a cell type

**Fig. 6:**
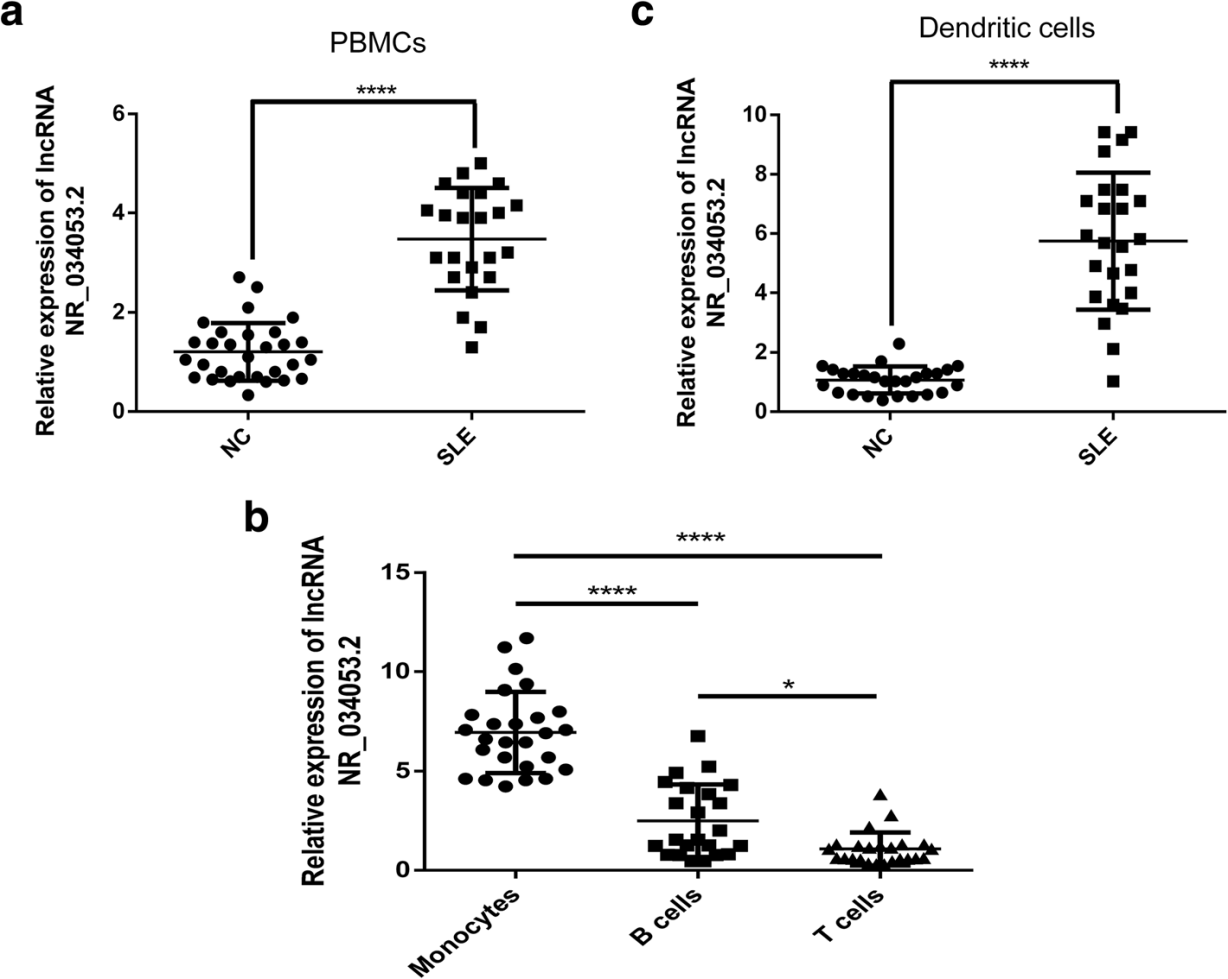
The relative expression of lncRNA NR_034053.2 was determined by qPCR in SLE patients and healthy donors (NC). **a** Expression of lncRNA NR_034053.2 in PBMCs of SLE patients and healthy donors (NC) was determined by qPCR analysis. **b** Expression of lncRNA NR_034053.2 in monocytes, B cells, and T cells from SLE patients. **c** lncRNA NR_034053.2 expression was upregulated in DCs of SLE patients compared with healthy donors. *P < 0.05, ****P < 0.0001

DCs from SLE patients produce much higher levels of the IFN-α. PBMCs from SLE patients (as compared with healthy donors) exhibited differential expression of lncRNAs due to a change in the number of a cell type (e.g., lymphocytes, monocytes, and DCs) in the peripheral blood. So, we performed tests with lncRNA NR_034053.2 as an example; the level of lncRNA NR_034053.2 expression was determined in PBMCs obtained from SLE patients and healthy controls by qRT-PCR analysis. As shown in Fig. 6a, increased lncRNA NR_034053.2 expression was detected in the PBMCs from SLE patients compared with that from healthy donors (*P* < 0.0001). To determine the cellular specificity of lncRNA NR_034053.2 expression, we examined the expression of lncRNA NR_034053.2 in the main subsets of PBMC (T cells, monocytes, and B cells) from SLE patients. LncRNA NR_034053.2 was expressed at substantially increased levels in monocytes compared with T and B cells (*P* < 0.0001) (Fig. 6b). In addition, DCs from PBMC of SLE patients showed notably enriched lncRNA NR_034053.2 expression as compared with healthy controls (*P* < 0.0001) (Fig. 6c), implying that the DCs are the primary lncRNA NR_034053.2-expressing cell type.

### LincRNA–mRNA expression pairs were built using 23 lincRNAs and 353 mRNAs

Thirty differentially expressed lincRNAs and 4101 differentially expressed mRNAs were analyzed through a construction of a coexpression network. The network was built using 23 lincRNAs and 353 mRNAs, forming 3784 lncRNA–mRNA expression pairs; of which, 2652 had a positive correlation, 1130 had a negative correlation, and 1 mRNA can bind to 1–17 lincRNAs. One lincRNA can bind to 16–284 mRNAs. For example, ENST00000339092.2 could be expressed with 284 upregulated mRNAs. We found that 62 coexpressed mRNAs were involved in KEGG pathway changes. As shown in Additional file 5: Figure S2, Additional file 6: Figure S3, and Additional file 7: Figure S4, the lincRNA–mRNA coexpression network participates in pathways related to lupus (e.g., 23 differential lincRNAs and 23 differential mRNAs participate in metabolic pathways). In the p53 signaling pathway, 3 mRNAs interact with 17 lincRNAs. In the FoxO signaling pathway, 11 lincRNAs can bind to 3 mRNAs and, in addition, can be combined with the lncRNA target gene prediction above.

### The ceRNA network was predicted using 9 validated lncRNAs, 15 miRNAs, and 155 mRNAs

**Fig. 7:**
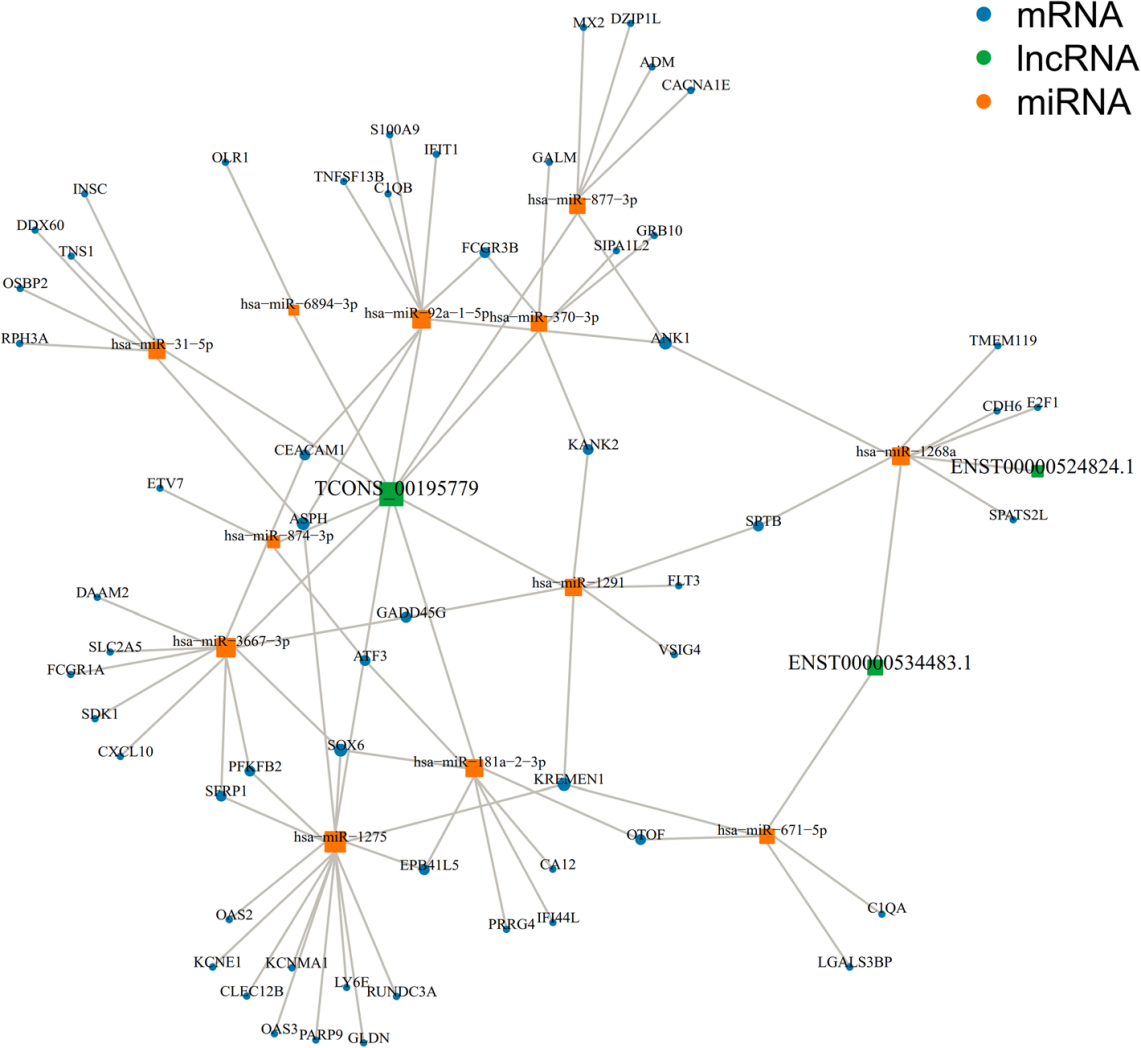
The mapping network of lncRNA-miRNA-mRNA interactions between 3 upregulated lncRNAs, 12 downregulated miRNAs, and 57 upregulated mRNAs

Through bioinformatics software prediction, we constructed regulatory networks among lncRNAs, miRNAs, and mRNAs by using 7 validated lincRNAs and 2 novel lncRNAs. miRanda, PITA, and RNAhybrid were used to predict differentially expressed miRNAs that may bind to lncRNAs. Two or more software consensus predictions were used as candidate miRNAs. We identified 9 differential lncRNAs and 41 differential miRNAs that formed 54 lncRNA–miRNAs (Additional file 8: Figure S5). To narrow the scope and facilitate functional research, we selected 40 miRNAs based on the expression level and sequenced the multiple of the 102 differential miRNAs. Several miRNAs (such as miR-31-5p [24]) have been studied in SLE (cluster analysis, Additional file 9: Figure S6). By comparing the 40 miRNAs with the candidate miRNAs, we identified 7 differential lncRNAs and 15 differential miRNAs, which formed 19 lncRNA–miRNA pairs. We then used miRDB, miRTarBase, miRWalk, and TargetScan software to predict differentially miRNAs that can bind to miRNAs. We also used 3 or more software programs to jointly predict the results of the target gene. The ceRNA network contained 7 validated lncRNAs (3 upregulated and 4 downregulated), 15 miRNAs (3 upregulated and 12 downregulated), and 155 mRNAs (57 upregulated and 98 downregulated), with a total of 304 pairs of lncRNA–miRNA–mRNA predicted. The numbers of pairs between the 3 upregulated lncRNAs (ENST00000524824.1, ENST00000534483.1, TCONS_00195779) and the downregulated miRNAs were 1, 2, and 10, respectively, corresponding to 6 and 10 downregulated mRNAs and 64 upregulated mRNAs (Fig. 7). The numbers of pairs between the 4 downregulated lncRNAs (ENST00000566788.1, ENST00000577528.1, ENST00000596960.1, and TCONS_00027049) with the upregulated miRNAs were 2, 1, 1, and 2 respectively corresponding to 98, 3, 25, and 98 downregulated mRNAs (Additional file 10: Figure S7). LncRNAs, miRNAs, and mRNAs can form a regulatory network through interaction and restricting 1 another in the gene network to participate in the physiological and pathological processes of SLE.

## Discussion

SLE is a common autoimmune disease that is characterized by excessive activation of T and B lymphocytes and increased production of autoantibodies and involves many systems or organs (such as the skin, kidney, blood, nervous system). Although the use of immunosuppressive agents and new therapeutic interventions has increased the survival rate of patients with SLE over the past 10 years, some patients died due to severe disease activity in the internal organs [25]. Relapse and individualized treatment are key issues in controlling lupus. However, the key aspects of immune dysfunction and the pathogenesis of SLE remain unclear.

Abnormalities in epigenetic mechanisms play an important role in the occurrence and development of SLE. In the past, noncoding transcripts were considered to be “noise” in transcription due to their lack of or low coding capacity [26]. However, subsequent studies indicated that 98% of ncRNAs in DNA transcripts play an important role in epigenetic modifications, particularly in gene regulation and maintaining physiological processes, such as cell and tissue homeostasis. The most well-known ncRNAs are microRNAs (miRNAs), which are 21–23 nt long and have been identified as an important regulator of SLE-related signaling pathways and genes [27], such as miR-146a, miR-155 [28], and miR-142-3p/5p [29]. Moreover, lncRNAs, which account for 80% of ncRNAs, are considered the star molecules following microRNAs and could influence the occurrence and development of diseases at the gene, transcription, and protein levels. LncRNAs exhibit specific temporal and spatial expression, and most of them show fine subcellular localization. LncRNAs are widely expressed in macrophages, monocytes, dendritic cells (DCs), neutrophils, T cells, and B cells and are closely related to their development, differentiation, and activation. In recent years, the roles of lncRNAs in autoimmune diseases have been widely reported. Manama et al. found that the expression levels of Gas5 in peripheral blood B cells and CD4+ T cells were significantly downregulated in patients with SLE; as such, Gas5 can inhibit the proliferation of T cells [30, 31]. LncRNA NEAT1 is mainly expressed in human monocytes; the expression of lncRNA NEAT1 is significantly upregulated in patients with SLE and positively correlated with the severity of the disease. As such, lncRNA NEAT1 can mediate inflammatory responses through the MAPK pathway [32]. LncRNA MEG3 was found to be involved in the pathogenesis of OA by regulating vascular endothelial growth factor [33]. LncRNA HOTAIR was considered a biomarker for the diagnosis of RA; this molecule was highly expressed in PBMCs and serum exosomes of patients with RA and promoted the extensive migration of activated macrophages [34]. Aberrant lncRNA expression, single-nucleotide polymorphisms, or base mutations at the corresponding positions are related to the occurrence and progression of autoimmune diseases.

Although few studies have been reported on lncRNAs in the past 20 years, the development of chips and high-throughput sequencing technologies has made it possible to identify lncRNAs on a large scale at the whole-genome level. Scholars have used chip technology to screen differential lncRNAs in SLE; the results promoted an in-depth study of the involvement of lncRNAs in the pathogenesis of lupus. However, the research results are inconsistent, and the expression levels obtained are contradictory. As a high-throughput transcriptome detection method based on second-generation sequencing, RNA-Seq has become the most promising technique because it can detect and quantify lncRNA expression without bias. The advantages of this technique include its ability to provide a highly dynamic range of expression and its superiority to chip analysis in terms of sensitivity and accuracy; Moreover, this technique does not need to predict the genomic locus of the transcribed region. At present, high-throughput sequencing of lncRNAs has been conducted in studies of tumors, such as colorectal [35] and liver cancers [36]; however, lncRNA sequencing of SLE has not been reported yet. Therefore, in this study, we use second-generation full-transcriptome high-throughput sequencing to detect differences in the expression levels of lncRNAs, miRNAs, and mRNAs in patients with lupus and healthy people. These three molecules were combined to analyze the interaction mechanism between lncRNAs and miRNAs as well as their interaction with mRNAs in SLE.

The sequencing results showed significant differences in the expression levels of 1087 lncRNAs (141 upregulated and 946 downregulated lncRNAs), 4101 mRNAs (770 upregulated and 3331 downregulated mRNAs; ∣log2(fold_change)∣ > 1, *q* < 0.001), and 102 upregulated miRNAs (39 upregulated and 63 downregulated miRNAs;∣log2(fold_change)∣ > 1, *q* < 0.05). Among which, those with multiples of more than fivefold include 47 lncRNAs (maximum upregulation multiple of 38.11160073 and maximum downregulation multiple of 66.77770783), 47 miRNAs (maximum upregulation multiple of 142.82 and maximum downregulation multiple of 153.11), and 306 mRNAs (maximum upscaling multiple of 6700.53 and maximum downregulation multiple of 69.07). Of the differentially expressed lncRNAs, 184 belong to lincRNAs (16.93%) and 220 belong to antisense lncRNAs (20.24%). We also discovered 5029 novel lncRNAs by annotation; of which, 132 had significant differences (77 upregulated and 55 downregulated lncRNAs). The expression levels of miR-142-3p (downregulated) and miR-31-5p (downregulated) are consistent with those reported in previous studies. Among lncRNAs, the GAS5 expression decreased in the sequencing results (fold change down = 2.075, *q* = 5.89 × 10^−80^). The lncRNA NEAT1 was significantly downregulated in patients with SLE, with a downregulation multiple of 1.498 and *q* = 8.882 × 10^−16^. Furthermore, qPCR results showed that the expression of seven lincRNAs and two novel lncRNAs was consistent with the sequencing results; however, no significant difference was found in the expression of ENST00000542819.1. These results indicate the reliability of the sequencing results. We also found some of the differentially significant miRNAs in other diseases; these miRNAs include miR-135a-5p (up = 57.46) [37], miR-138-5p (down = 33.25) [38], miR-370-5p (down = 71.00) [39], and miR-490-3p (up = 7.26) [40], which may participate in the pathogenesis of SLE. The abovementioned lncRNAs and miRNAs with significant differences may serve as clinic markers for the diagnosis and evaluation of SLE activities and therapeutic effects.

To further clarify the mechanism of lncRNA in SLE, we selected the most studied and functional lincRNAs. Long intergenic noncoding RNAs (lincRNA) are a type of lncRNA that is transcribed from the gap between adjacent protein-coding genes. In lincRNAs, no overlap exists between the genomic region and the coding gene, and no coding gene exists within 30 kb in upstream and downstream [41, 42]. The human body comprises thousands of lincRNAs, but only the functions of less than 1% have been identified [43]. SLE is a complex multi-gene disease. The completion of the Human Genome Project indicates that multiple loci are associated with SLE, and the genetic linkage has been confirmed with obvious correlations including 1q23-24, 1q31-32, 2p35-37, 4p16-15.2, 6p21-11, and 16q12-13 [44]. Therefore, we focused on lincRNAs located at the loci of SLE susceptibility and screened 30 lincRNAs combined with the expression quantity (RPKM) and multiples of diversity. For example, ENST00000524824.1, located at 11p15.5, where IRF7 is also located, may participate in the TLR/IFN signaling pathway [45]. ENST00000601116.1 and ENST00000556030.1 are located in 19q13.2 [46] and 15q26.2 [44], respectively, which are considered to be lupus gene susceptibility loci. ENST00000417522.1 (1q32.1) is associated with IL10 and involved in the B cell signaling pathway [15]. ENST00000577528.1 (1p36.11) is associated with IL28RA and involved in the T cell signaling pathway [47].

Many lincRNAs coexpress with nearby coding genes and play an important role in transcriptional activation and phylogeny [48]. In this study, we constructed a lincRNA–mRNA coexpression network to discover the relationship between differential lincRNAs and differential mRNAs. We found that 23 lincRNAs and 353 mRNAs in 30 lincRNAs were involved in the network formation, forming 3782 lncRNA–mRNA expression pairs; of which, ENST00000339092.2 can be coexpressed with up to 284 mRNAs. The function of lncRNAs can be mainly detected by analysis of the effect of its target gene. Many lncRNAs can affect the expression of genes encoded within their vicinity in a *cis*-acting manner or regulate the translation of genes by complementary base pairing [12]. Therefore, we predicted the above 30 lincRNA target genes. The results show that 23 lincRNAs may act on 704 differential target genes, forming 890 lncRNA–gene connections (Additional file 4: Figure S1), except ENST00000596960.1; 7 successfully validated lincRNAs have more likely target genes, of which ENST00000601116.1 have the most 270 target genes. In the qPCR results, the most significantly upregulated and downregulated ENST00000524824.1 and ENST00000577528.1 have 134 and 60 target genes, respectively. To further explore the biological functions of 23 lincRNAs, we conducted GO and pathway analyses on 740 differential target genes (Fig. 4). These genes were mainly enriched in the metabolic pathways, p53 signaling pathway, glycosphingolipid biosynthesis-globo series pathway, and FoxO signaling pathway, which are related to the pathogenesis of SLE. The metabolic abnormalities of lipids, proteins, and androgens in SLE have been widely reported. P53 is involved in the apoptosis and pathogenesis of SLE, and its expression level is significantly associated with SLE Disease Activity Index and the levels of anti-DNA antibody and IL-10 [19]. In glycosphingolipid biosynthesis-globo series, glycolipids and their derivatives play an important regulatory role in cell growth, differentiation, and apoptosis. Evidence also indicates that glycolipid molecules participate in the immune response process [20]. In the FoxO signaling pathway, FOX01 is negatively correlated with the activity of SLE [21]; the activation of FOX01 can stimulate the expression of Bim, which is a member of the pro-apoptotic Bcl-2 gene family and plays an important role in apoptosis. We found that 62 coexpressed mRNAs are involved in the KEGG pathway changes and that the lincRNA–mRNA coexpression network is involved in the above pathways (Additional file 5: Figure S2, Additional file 6: Figure S3, Additional file 7: Figure S4). For example, 23 differential lincRNAs with 23 differential mRNAs are involved in metabolic pathways. In the p53 signaling pathway, 3 mRNAs interact with 17 lincRNAs. In the FoxO signaling pathway, 11 lincRNAs bind to 3 mRNAs. Based on the combined results of lincRNA–mRNA coexpression analysis and target gene prediction, 36 mRNAs were coexpressed with 1 of the 7 successfully validated lincRNAs and were predicted to be the corresponding lincRNA target genes. Forty-four pairs of lincRNA–mRNA were constructed. Some of the 36 mRNAs were confirmed to be one of the key genes in the pathogenesis of SLE. Interferon-induced protein with tetratricopeptide repeats 3 (IFIT3) is one of the genes which contribute to the overactive cGAS-STING signaling pathway in SLE monocytes, which may serve as a therapeutic target to block the production of type I IFN and other pro-inflammatory cytokines by cGAS-STING signaling pathway [49]. IFITM3 is a family of interferon-inducible transmembrane proteins (IFITM) that play a role in several biological activities, such as interferon homotypic cell adhesion and cell antiproliferative activity [50]. The expression level of IFITM3 was significantly higher in patients with SLE and was significantly negatively correlated with complement C3 and C4, which may be involved in the pathological process of SLE. IFIT3 (up = 3.65) and IFITM3 (up = 5.09) are the target genes of 3 lincRNAs (ENST00000524824.1, ENST00000531076.1, and ENST00000534483.1) and are coexpressed with them. Hence, these three lincRNAs may play an important role in the pathogenesis of SLE and affect disease activity by participating in the interferon (IFN) family and their immune regulatory pathways.

SLE in patients exhibits a strong gender bias. Was the differential expression of lncRNAs in our screen gender-dependent? TSIX, known as ENST00000604411.1 or LINC00013, expresses a noncoding antisense transcript across the 3′ end of the XIST locus. It also protects the active-X from ectopic silencing once X-inactivation has commenced [23]. There is an increased incidence and prevalence of systemic lupus erythematosus in females, which might involve X chromosome inactivation [22]. In our study, we found that not only the expression of TSIX in SLE patients was significantly higher than that in healthy donors, but also the expression of TSIX in SLE female patients was significantly higher than that in SLE male patients. Therefore, the upregulated TSIX might facilitate X chromosome inactivation through protecting the active-X from ectopic silencing and take part in the pathogenesis of SLE.

Whether SLE patients exhibited a robust or weak “IFN signature”? Further, whether a particular SLEDAI score associated with the “IFN signature” or not? In recent years, research on the imbalance of cytokine networks in SLE has become increasingly deep, while IFN-α is an important regulator of the body’s inflammation and immune response. In our study, the IFN-α level in the serum of SLE patients was higher than that in healthy donors, and the change was positively correlated with the SLE Disease Activity Index (SLEDAI), suggesting that IFN-α may play an important role in the SLE pathogenesis because it can activate autoreactive T and B lymphocytes and induce B cells to produce antibodies. In addition, IFN-α can also induce the maturation and division of dendritic cells (DCs) and enhance the ability of DCs in capturing and presenting autoantigens, thereby breaking tolerance and accelerating the occurrence and development of SLE [51].

Whether PBMCs from SLE patients (as compared with healthy donors) exhibited differential expression of lncRNAs due to a change in the number of a cell type (e.g., lymphocytes, monocytes, and DCs) in peripheral blood? In most SLE patients, the expression of type-I IFN regulatory genes, also known as IFN signal, increased [52]. Some target genes of differentially expressed lncRNA in moDCs were associated with the type-I IFN system, such as IRF5. IRF5 is a target gene of lncRNA NR_034053.2 and is associated with an increase in IFN activity in the blood of SLE patients [53]. The increase in expression level of lncRNA NR_034053.2 in the moDCs of SLE patients may suggest that moDCs play a regulatory role in the production of lupus type I IFN [54]. Our results showed that, for PBMC and DCs isolated from the same volume of the whole blood of SLE patients and healthy donors, the expression levels of lncRNA NR_034053.2 in SLE patients were significantly higher than those in healthy donors, and for the T cells, B cells, and monocytes isolated from the same volume of the whole blood of SLE patients, the expression level of lncRNA NR_034053.2 in monocytes was also significantly higher than that in T cells and B cells. These results might suggest PBMCs from SLE patients (as compared with healthy donors) exhibited differential expression of lncRNAs due to a change in the number of a cell type (e.g., lymphocytes, monocytes, and DCs) in the peripheral blood.

In 2011, Salmena et al. proposed competing endogenous RNA (ceRNA) theory, which reveals a new mechanism of RNA interaction [55]. ceRNAs can competitively bind to miRNAs through the same microRNA response elements (MREs), thereby affecting gene silencing caused by miRNA binding to mRNA to regulate gene expression [56]. In recent years, the function of lncRNAs as ceRNA-regulated gene expression has been elucidated in many diseases. Linc-MD1 binds to miR-133 and miR-135 through complementary base pairing and competitively inhibits their binding to target genes, thereby regulating muscle differentiation [57]. In colorectal cancer, lncRNA UICLM is a competitive endogenous RNA (ceRNA) that binds to miR-215 and suppresses the inhibition of ZIR2 by miR-215, thereby playing a role in promoting liver metastasis of colon cancer [58]. In view of the discovered lncRNAs, miRNAs, and other ncRNAs can participate in the biological processes of SLE occurrence and development. We can also infer the important relationship between ceRNA network imbalance and SLE disease. Through prediction of bioinformatics software, we constructed a ceRNA regulatory network among differential lncRNAs, miRNAs, and mRNAs. The ceRNA network contained 7 successfully validated significantly differential lincRNAs (3 upregulated and 4 downregulated), 15 significantly differential miRNAs (3 upregulated and 12 downregulated), and 155 significantly differential mRNAs (57 upregulated and 98 downregulated), forming 304 pairs of lncRNA–miRNA–mRNA. TCONS_00195779 (up = 4.39) can bind up to 10 miRNAs and form 64 lncRNA–miRNA–mRNA connections. miR-31-5p was found to be underexpressed in T cells of patients with SLE; this molecule promotes the inhibition of the target gene RhoA and the downregulation of IL-2 expression and thus participates in immune disorders of SLE [24]. We can speculate that TCONS_00195779-miR-31-5p-RhoA may be involved in the pathogenesis of lupus. In addition, miR-1268a (down = 2.95), miR-143-3p (up = 2.07), and miR-182-5p (up = 2.13) can bind to 2, 3, and 2 lncRNAs, respectively, and could be the key nodes in the ceRNA network. In the target genes, we also found some susceptibility genes with SLE. IFIT1 and the abovementioned IFIT3 are the representative genes of the type I interferon system. The upregulated IFIT1 in lupus plays an important role in the production of cytokines IL-4 and IL-10, which may be related to the imbalance of Th1/Th2. CXCL10, which belongs to the chemokine family, is significantly upregulated in SLE serum. CXCL10 can induce the chemotactic migration of macrophages, neutrophils, lymphocytes, and monocytes and mediate the aggregation and activation of leukocytes at the site of inflammation [59]. MALT1, a regulatory protein in the NF-κβ signaling pathway, plays an important role in the activation of NF-κβ mediated by T cell receptor (TCR) and the regulation of lymphocyte proliferation and differentiation [60]. This protein was found to be significantly underexpressed in the PBMCs of patients with SLE [61], consistent with our sequencing results. Hence, we speculate that TCONS_00195779-miR-92a-1-5p-IFIT1 (up-down-up), TCONS_00195779-miR-3667-3p-CXCL10 (up-down-up), and ENST00000566788.1-miR-182-5p-MALT1 (down-up-down) are a possible SLE-related lncRNA–miRNA–mRNA axis.

In summary, research on lncRNA in SLE has made some progress but is still in its infancy stage; related studies have focused on the discovery of differential lncRNAs.

## Conclusions

In this study, we use full high-throughput sequencing to analyze the differences in the expression profiles of lncRNAs, miRNAs, and mRNAs in the PBMCs of patients with SLE at the genome-wide level. By using the gene loci and gene information of differentially expressed lncRNAs and bioinformatics analysis methods, we can select lincRNAs that may be closely related to SLE and preliminary ascertain their possible functions. We also further made experiments to confirm the following: (1) the differential expression of lncRNAs may be gender-dependent in SLE patients, (2) a positive correlation between IFN-α and disease activity in SLE patients, and (3) PBMCs exhibiting differential expression of lncRNAs may be due to a change in the number of a cell type. The results provide a foundation for clarifying the mechanism of lncRNAs in the pathogenesis of SLE and impart new ideas for effective treatment of SLE.

## Additional files

Additional file 1: **Table S1.** Primer sequence for q-PCR. (DOCX 14 kb)

Additional file 2: **Table S2.** Fifteen significantly upregulated lincRNAs in SLE. (DOCX 12 kb)

Additional file 3: **Table S3.** Fifteen significantly downregulated lincRNAs in SLE. (DOCX 12 kb)

Additional file 4: **Figure S1.** Target gene prediction network map of 23 lincRNAs from 18 genes. (TIF 5716 kb)

Additional file 5: **Figure S2.** Twenty-three lincRNAs interacted with 23 mRNAs in the Metabolic pathways. (TIF 2422 kb)

Additional file 6: **Figure S3.** Seventeen lincRNAs interacted with 3 mRNAs in the meaningful p53 signaling pathway. (TIF 2190 kb)

Additional file 7: **Figure S4.** Eleven lincRNAs interacted with 3 mRNAs in the meaningful FoxO signaling pathway. (TIF 1765 kb)

Additional file 8: **Figure S5.** The mapping network of lncRNA-miRNA interactions between 9 dysregulated lncRNAs and 41 dysregulated miRNAs. (TIF 2488 kb)

Additional file 9: **Figure S6.** The cluster heat maps of 40 screened miRNAs:40 miRNAs were selected based on expression level and sequenced the multiple of the 102 differential miRNAs for the cluster analysis. (TIF 2567 kb)

Additional file 10: **Figure S7.** The mapping network of lncRNA-miRNA-mRNA interactions between 4 downregulated lncRNAs, 3 upregulated miRNAs and 98 downregulated mRNAs. (TIF 2445 kb)

## Abbreviations

CAMs: Cell adhesion molecules
ceRNA: Competing endogenous RNA
CPC: Coding Potential Calculator
DCs: Dendritic cells
ELISA: Enzyme-linked immunosorbent assay
GO: Gene Ontology
IFIT3: Interferon-induced protein with tetratricopeptide repeats 3
IFITM: Interferon-inducible transmembrane proteins
KEGG: Kyoto Encyclopedia of Genes and Genomes
Linc RNAs: Long intergenic noncoding RNAs
Lnc RNAs: Long noncoding RNAs
miRNAs: MicroRNAs
MREs: MicroRNA response elements
PBMCs: Peripheral blood mononuclear cells
qRT-PCR: Real-time quantitative reverse transcriptase polymerase chain reaction
RT: Reverse transcription
SLE: Systemic lupus erythematosus
SLEDAI: Systemic Lupus Erythematosus Disease Activity Index
TCR: T cell receptor
