# Supplementary material for: Full high-throughput sequencing analysis of differences in expression profiles of long noncoding RNAs and their mechanisms of action in systemic lupus erythematosus
Source: Arthritis Res Ther. 2019 Mar 5;21:70. doi: 10.1186/s13075-019-1853-7 (PMC6402184; doi:10.1186/s13075-019-1853-7)
Supplement: Supplementary file 1 — Table S1. Primer sequence for q-PCR. (DOCX 14 kb) [file 13075_2019_1853_MOESM1_ESM.docx]

**Table S1:Primer sequence for q-PCR**

| Primer name | Sequence (5’ to 3’) |
| --- | --- |
| ENST00000524824.1 | F: TCCATTCTCAAACTGCTGTGAGTG |
|  | R: GTGTGTTACTTGGGAGTTCCAGC |
| ENST00000531076.1 | F: GCCTCCTCTGATTCTGCACC |
|  | R: GCACTCACAGCAGTTTGAGAATG |
| ENST00000534483.1 | F: GGCCTGTCTTCTTCTTCCTAAGT |
|  | R: CTGACCTAAGCTGATTTAAGGCAC |
| ENST00000542819.1 | F: AAGCTCTTCTTTGTCTTGTTCACCT |
|  | R: CCAAAATGTTACAGTGTCACTGCC |
| ENST00000596960.1 | F: GCCAGTTGATTCTGACAATTTCTC |
|  | R: CCTCCAGATCGAAGTATTTCAGC |
| ENST00000566788.1 | F: TGAGCTAATGTTGAGTGAGCAGG |
|  | R: CTGAGTGACTCACTGTTCCCAAG |
| ENST00000601116.1 | F: CTATGTTGAAAACAGGATGCATGAC |
|  | R: CAAACTCGGTTCATATTTCATCCAC |
| ENST00000577528.1 | F: CCTAAGTCAGTGCCATAACCTCAGT |
|  | R: TGGCTTTCTTAGAGGCTCAATC |
| TCONS_00195779 | F: AGAAAGGTCCCAACTGTAGACCAC |
|  | R: AAGAGACATGGTTTGACGAGTCC |
| TCONS_00027049 | F: TTTGCCAAATACATGGCTATTACTT |
|  | R: TTTGTGGTGTTAGAGACCACCAG |
| ENST00000604411.1 (TSIX) | F: AGCCCCACTTCACATTAGACC |
|  | R: TGATGTTGCAGTCCTGTGAGG |
| LncRNA NR_034053.2 | F: AGCCTGGATTTCAGTGGGTAG |
|  | R: GCCGGATCTGTAACAACTGGTC |
| GAPDH | F: TGACTTCAACAGCGACACCCA |
|  | R: CACCCTGTTGCTGTAGCCAAA |
