# Supplementary material for: Full high-throughput sequencing analysis of differences in expression profiles of long noncoding RNAs and their mechanisms of action in systemic lupus erythematosus
Source: Arthritis Res Ther. 2019 Mar 5;21:70. doi: 10.1186/s13075-019-1853-7 (PMC6402184; doi:10.1186/s13075-019-1853-7)
Supplement: Supplementary file 2 — Table S2. Fifteen significantly upregulated lincRNAs in SLE. (DOCX 12 kb) [file 13075_2019_1853_MOESM2_ESM.docx]

Table S2: Fifteen significantly upregulated lincRNAs in SLE

| 15 Up-regulated LincRNAs | | | |
| --- | --- | --- | --- |
| LincRNA name | Gene Symbol | log2(Fold_change) | q-value |
| ENST00000524824.1 | ENSG00000255328.1 | 1.996663 | 2.77E-15 |
| ENST00000531076.1 | ENSG00000255328.1 | 1.853572 | 0 |
| ENST00000534483.1 | ENSG00000255328.1 | 1.849280 | 0 |
| ENST00000605240.1 | ENSG00000270972.1 | 1.778215 | 0 |
| ENST00000602949.1 | ENSG00000270030.1 | 1.585967 | 1.73E-13 |
| ENST00000556030.1 | ENSG00000258476.1 | 1.557303 | 2.24E-09 |
| ENST00000514568.2 | ENSG00000249790.2 | 1.425672 | 2.77E-15 |
| ENST00000542819.1 | ENSG00000255801.1 | 1.303092 | 5.31E-15 |
| ENST00000594721.1 | ENSG00000268734.1 | 1.283079 | 5.31E-15 |
| ENST00000609268.1 | ENSG00000273272.1 | 1.257316 | 5.31E-15 |
| ENST00000422780.3 | ENSG00000249790.2 | 1.235551 | 2.77E-15 |
| ENST00000533082.1 | ENSG00000254789.1 | 1.228517 | 1.98E-09 |
| ENST00000540299.1 | ENSG00000249790.2 | 1.199568 | 2.77E-15 |
| ENST00000595748.1 | ENSG00000231412.2 | 1.081249 | 5.60E-05 |
| ENST00000609178.1 | ENSG00000272666.1 | 1.065154 | 1.15E-14 |
