# Supplementary material for: Full high-throughput sequencing analysis of differences in expression profiles of long noncoding RNAs and their mechanisms of action in systemic lupus erythematosus
Source: Arthritis Res Ther. 2019 Mar 5;21:70. doi: 10.1186/s13075-019-1853-7 (PMC6402184; doi:10.1186/s13075-019-1853-7)
Supplement: Supplementary file 3 — Table S3. Fifteen significantly downregulated lincRNAs in SLE. (DOCX 12 kb) [file 13075_2019_1853_MOESM3_ESM.docx]

Table S3: Fifteen significantly downregulated lincRNAs in SLE

| 15 Down-regulated LincRNAs | | | |
| --- | --- | --- | --- |
| LincRNA name | Gene Symbol | log2(Fold_change) | q-value |
| ENST00000577528.1 | ENSG00000264443.1 | -1.854757 | 2.33E-82 |
| ENST00000566942.1 | ENSG00000259834.1 | -1.491744 | 6.55E-164 |
| ENST00000587011.1 | ENSG00000267705.1 | -1.384547 | 1.03E-09 |
| ENST00000553983.1 | ENSG00000258800.1 | -1.378116 | 1.10E-07 |
| ENST00000417522.1 | ENSG00000240754.1 | -1.371315 | 0.000114972 |
| ENST00000554058.1 | ENSG00000258301.2 | -1.242548 | 4.66E-15 |
| ENST00000566788.1 | ENSG00000260539.1 | -1.206138 | 2.17E-69 |
| ENST00000553758.1 | ENSG00000258610.1 | -1.199655 | 1.22E-12 |
| ENST00000556072.1 | ENSG00000258301.2 | -1.184774 | 2.66E-23 |
| ENST00000602688.1 | ENSG00000270113.1 | -1.159310 | 2.99E-12 |
| ENST00000595395.1 | ENSG00000268027.1 | -1.149196 | 3.37E-09 |
| ENST00000601116.1 | ENSG00000268027.1 | -1.120020 | 8.65E-28 |
| ENST00000339092.2 | ENSG00000188971.4 | -1.081902 | 1.41E-89 |
| ENST00000596960.1 | ENSG00000268544.1 | -1.020343 | 4.42E-15 |
| ENST00000593427.1 | ENSG00000268205.1 | -1.005223 | 4.46E-46 |
