# Supplementary figures and images for: Full high-throughput sequencing analysis of differences in expression profiles of long noncoding RNAs and their mechanisms of action in systemic lupus erythematosus
Source: Arthritis Res Ther. 2019 Mar 5;21:70. doi: 10.1186/s13075-019-1853-7 (PMC6402184; doi:10.1186/s13075-019-1853-7)

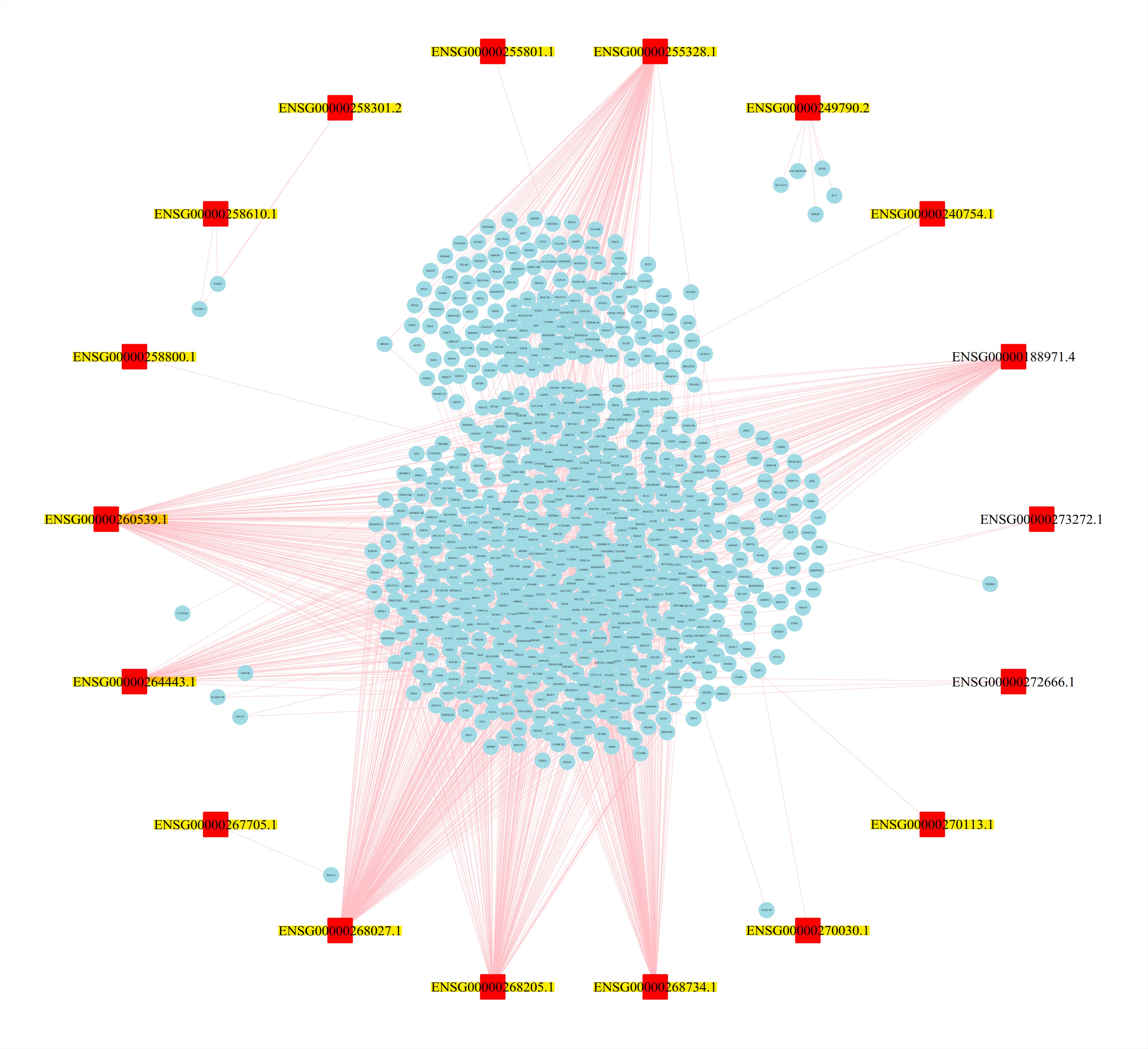

Supplement: Supplementary file 4 — Figure S1. Target gene prediction network map of 23 lincRNAs from 18 genes. (TIF 5716 kb) [file 13075_2019_1853_MOESM4_ESM.tif]

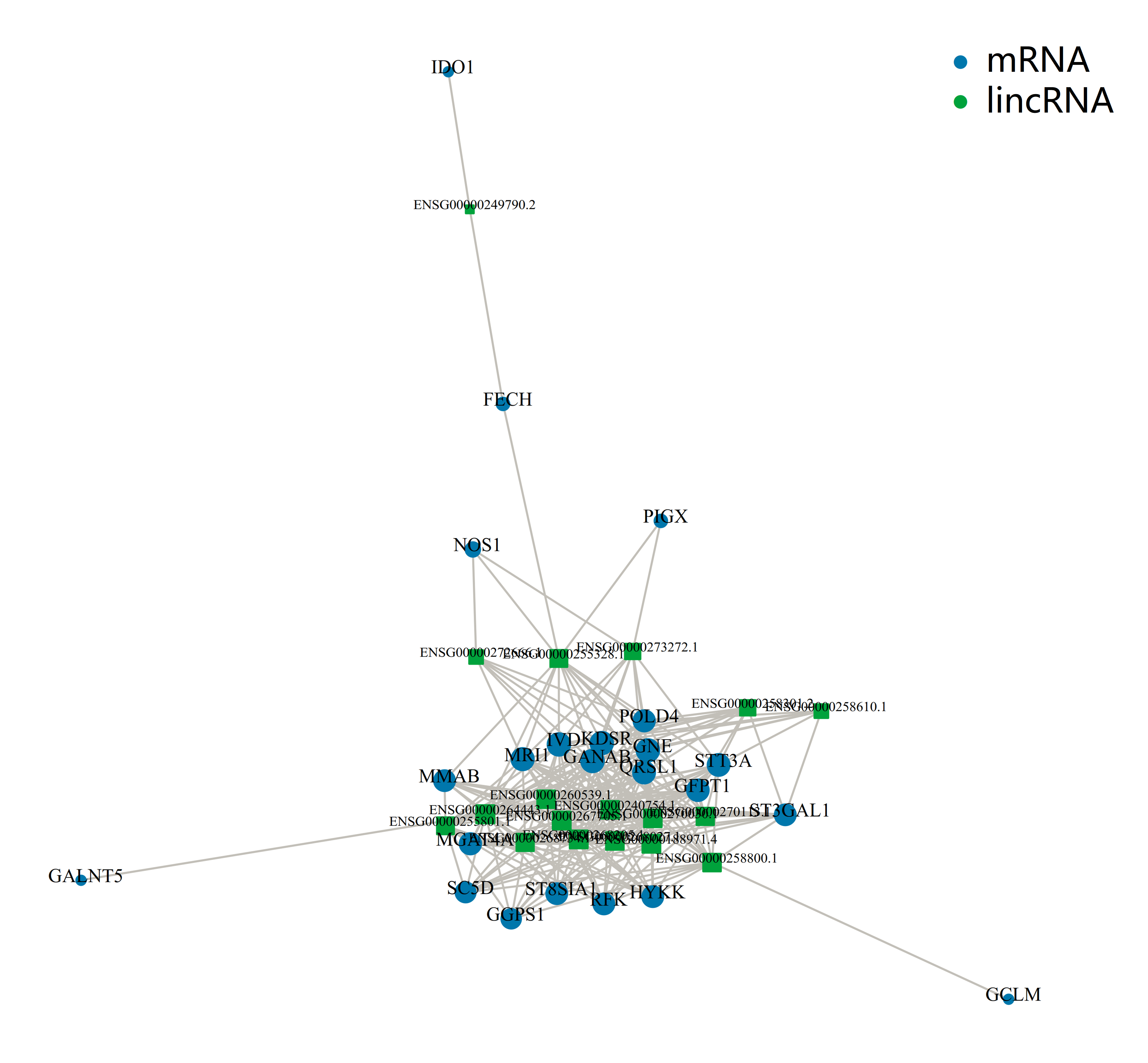

Supplement: Supplementary file 5 — Figure S2. Twenty-three lincRNAs interacted with 23 mRNAs in the Metabolic pathways. (TIF 2422 kb) [file 13075_2019_1853_MOESM5_ESM.tif]

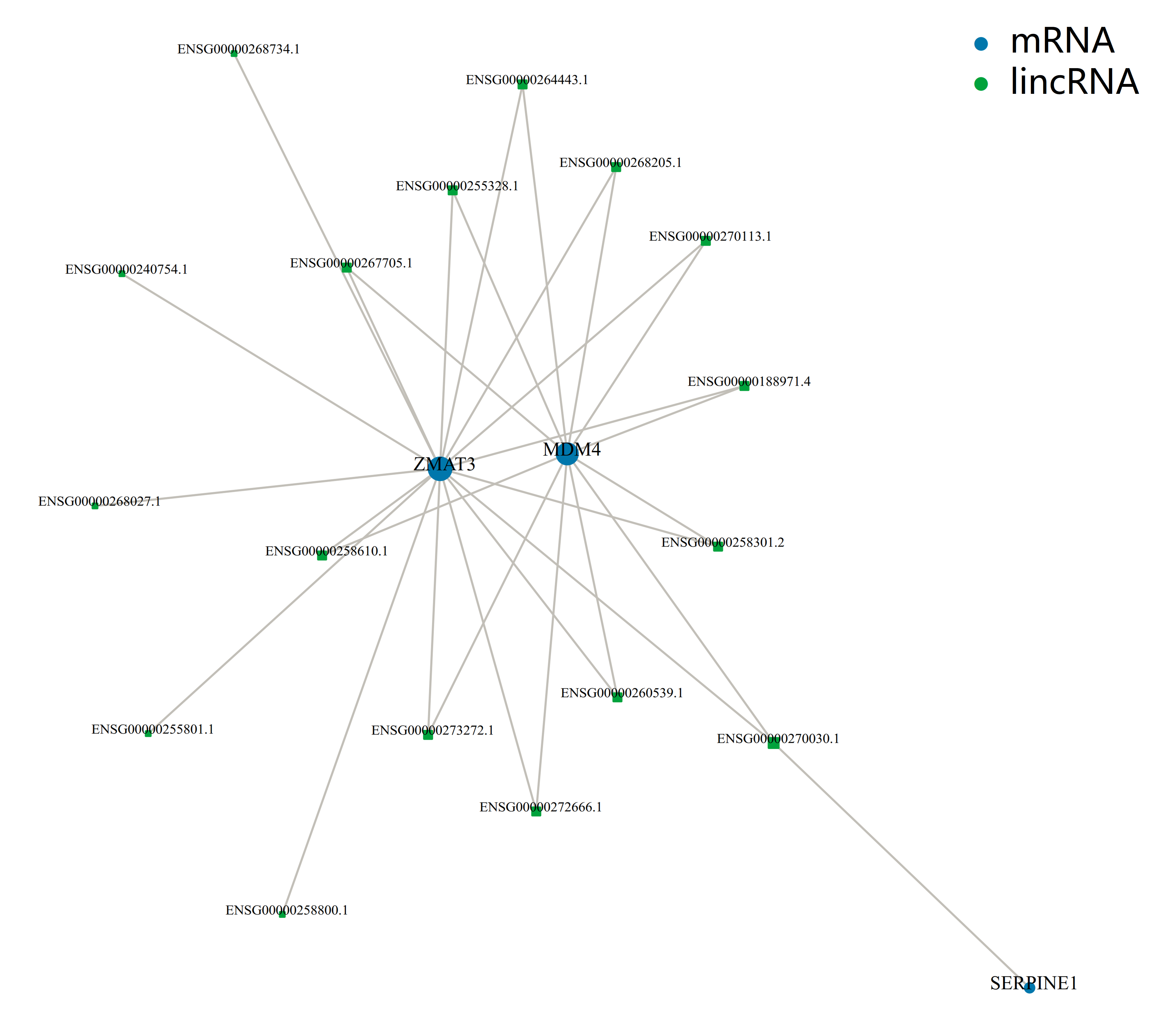

Supplement: Supplementary file 6 — Figure S3. Seventeen lincRNAs interacted with 3 mRNAs in the meaningful p53 signaling pathway. (TIF 2190 kb) [file 13075_2019_1853_MOESM6_ESM.tif]

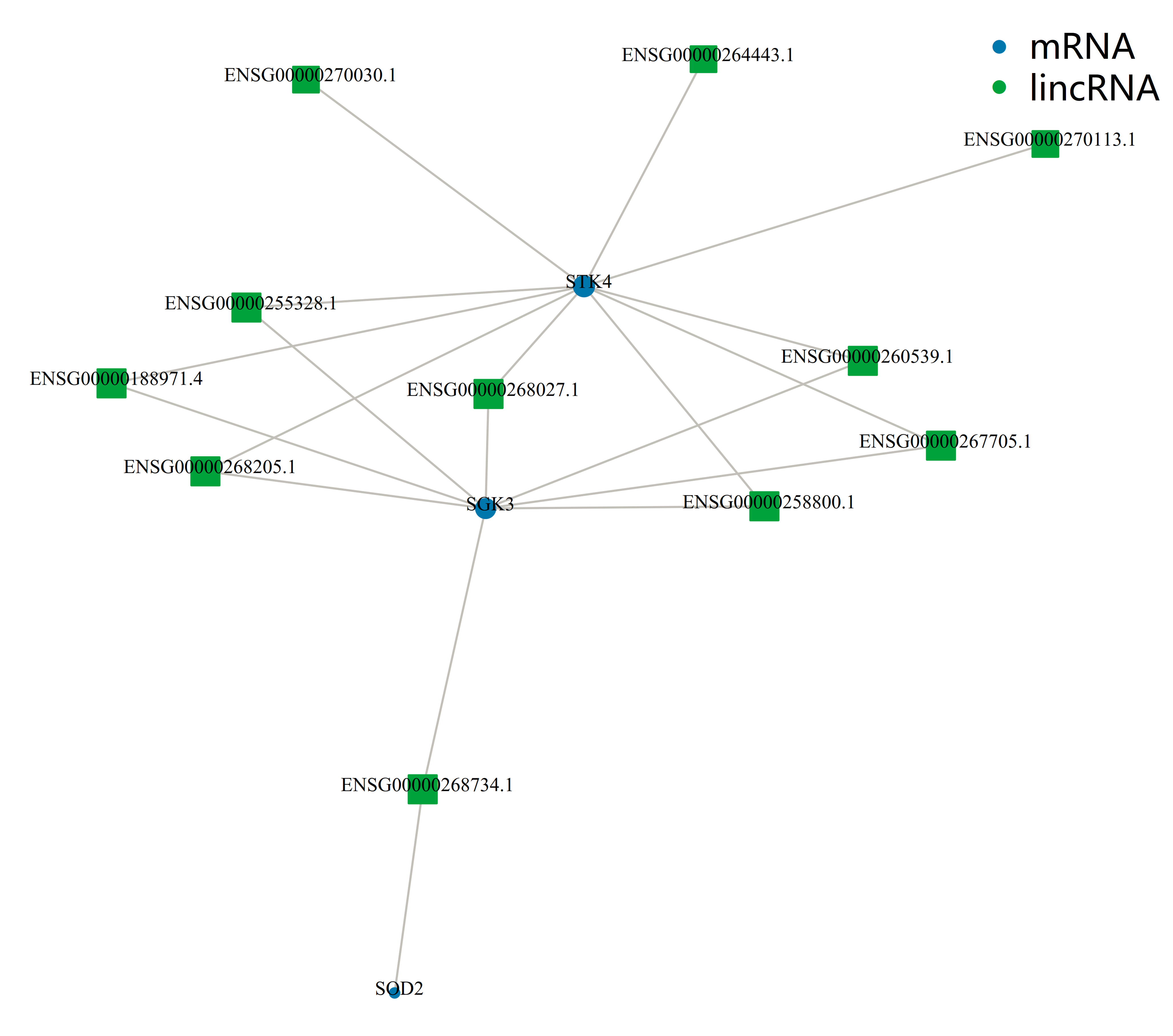

Supplement: Supplementary file 7 — Figure S4. Eleven lincRNAs interacted with 3 mRNAs in the meaningful FoxO signaling pathway. (TIF 1765 kb) [file 13075_2019_1853_MOESM7_ESM.tif]

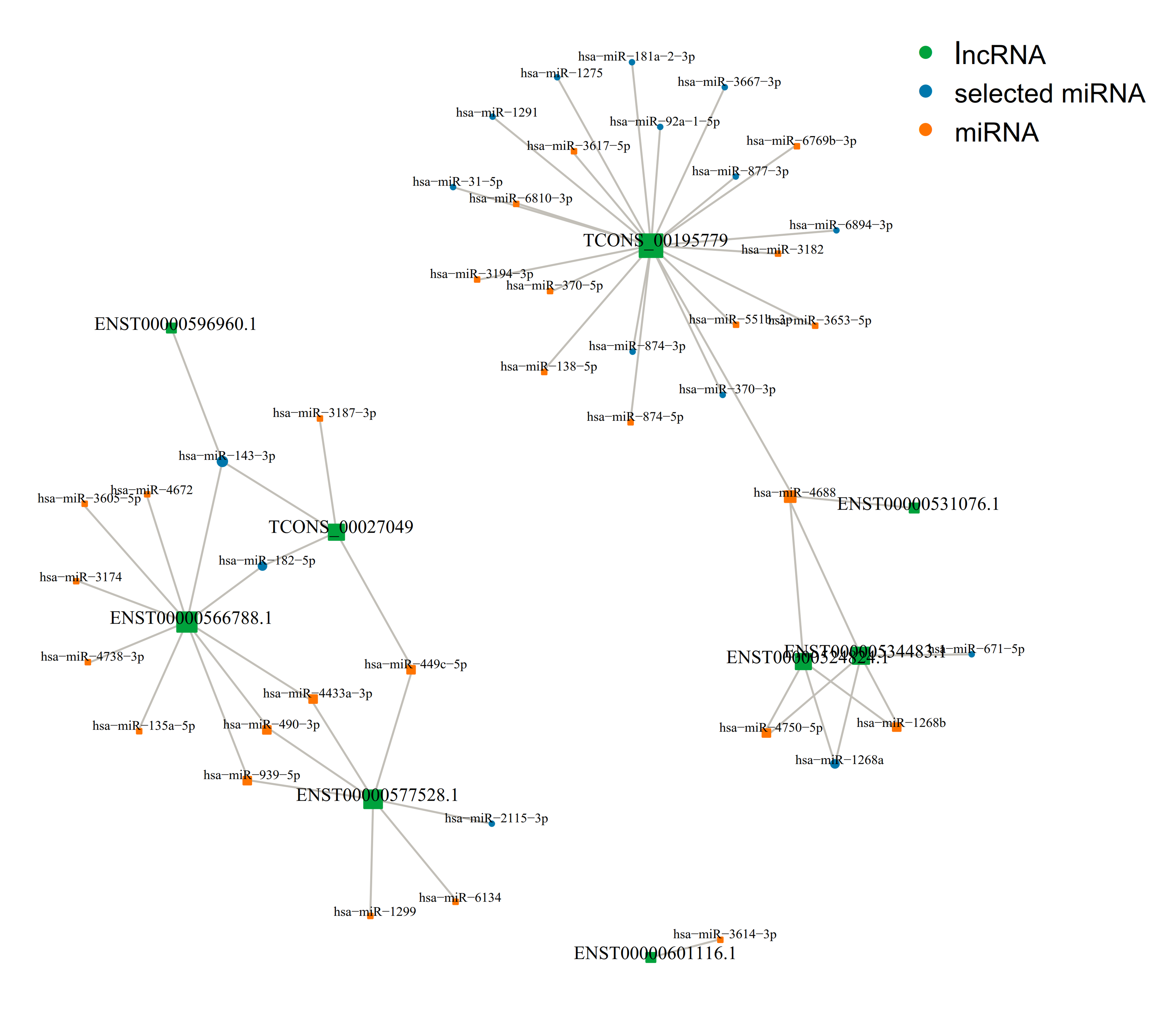

Supplement: Supplementary file 8 — Figure S5. The mapping network of lncRNA-miRNA interactions between 9 dysregulated lncRNAs and 41 dysregulated miRNAs. (TIF 2488 kb) [file 13075_2019_1853_MOESM8_ESM.tif]

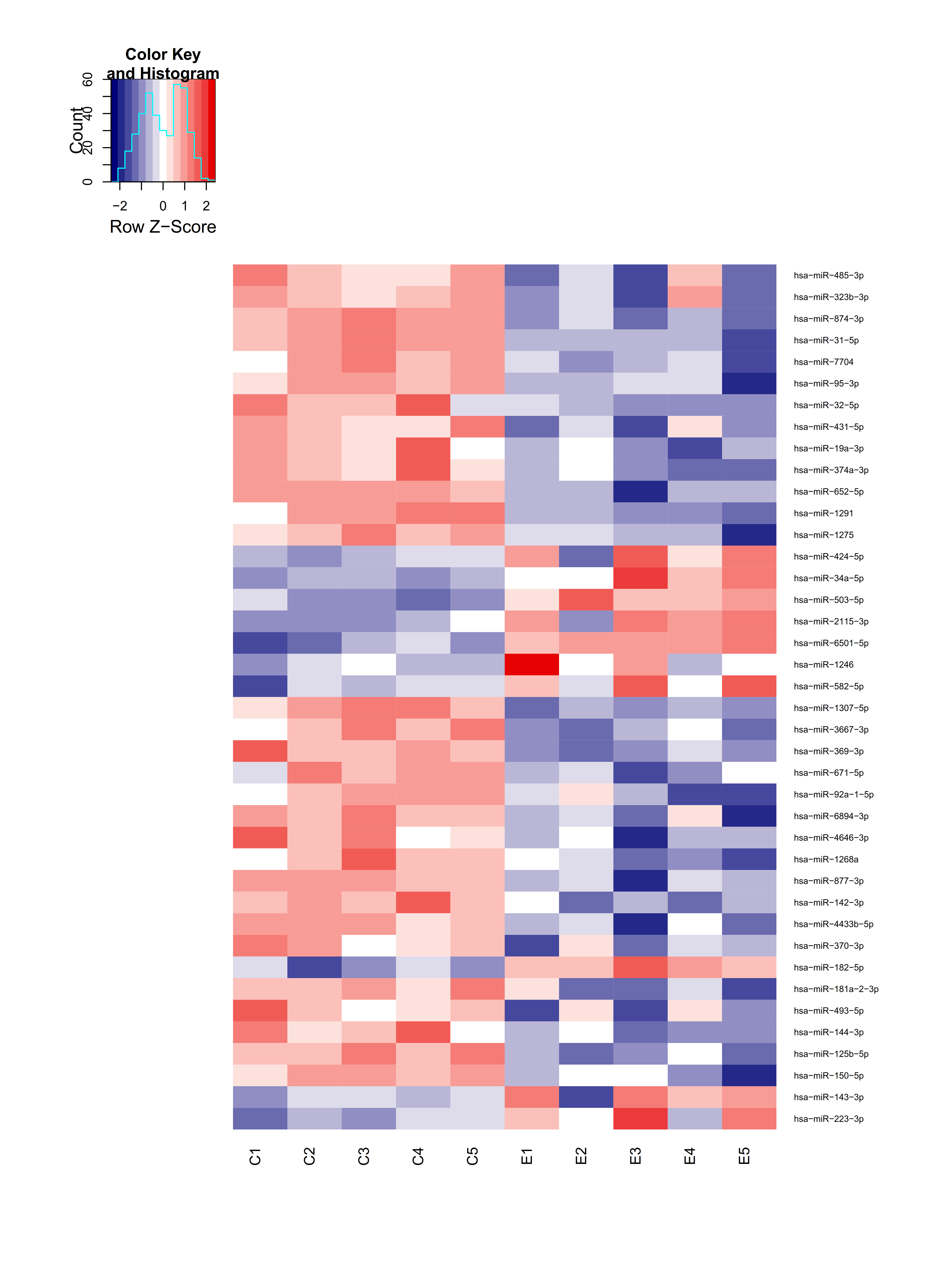

Supplement: Supplementary file 9 — Figure S6. The cluster heat maps of 40 screened miRNAs:40 miRNAs were selected based on expression level and sequenced the multiple of the 102 differential miRNAs for the cluster analysis. (TIF 2567 kb) [file 13075_2019_1853_MOESM9_ESM.tif]

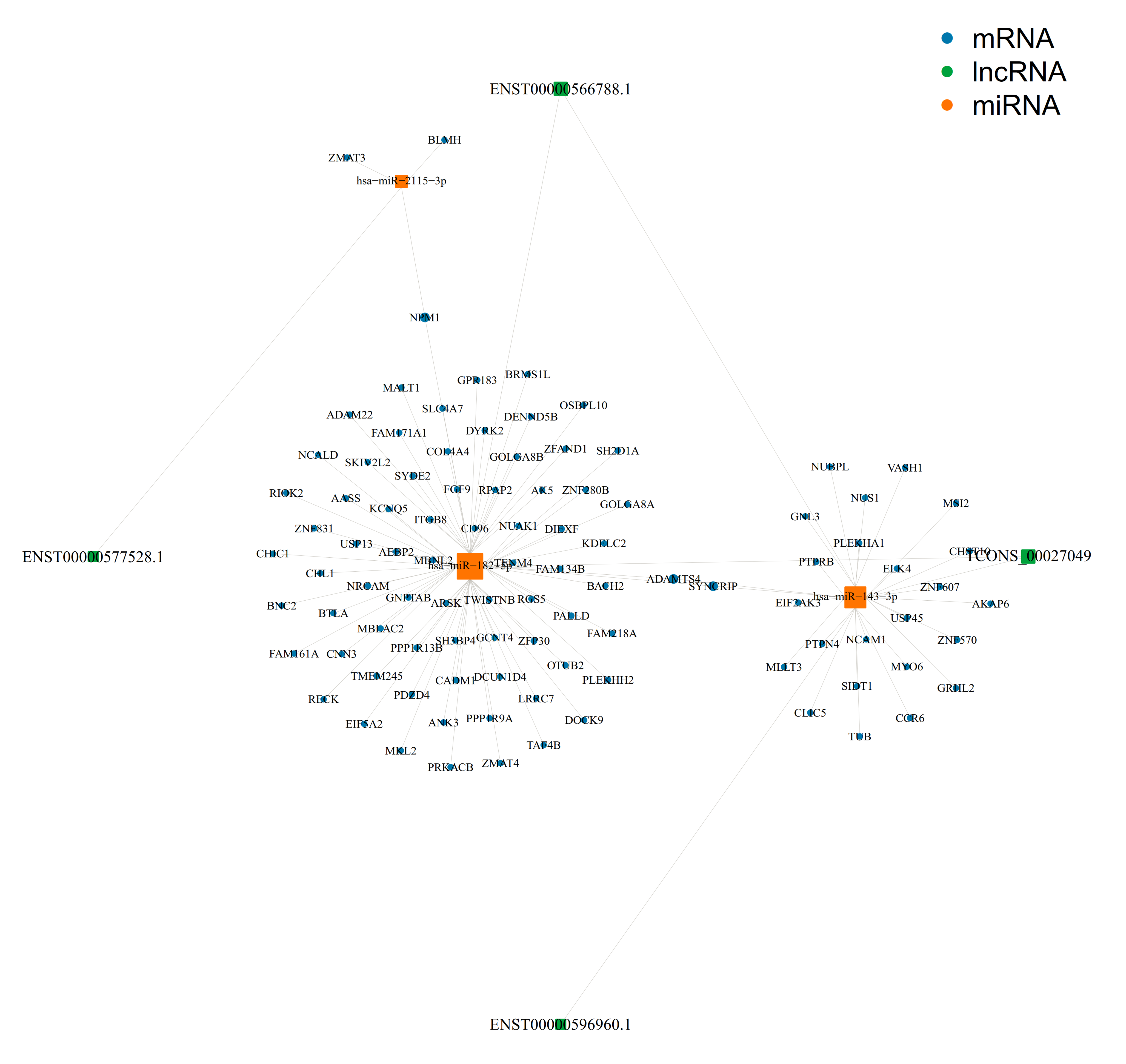

Supplement: Supplementary file 10 — Figure S7. The mapping network of lncRNA-miRNA-mRNA interactions between 4 downregulated lncRNAs, 3 upregulated miRNAs and 98 downregulated mRNAs. (TIF 2445 kb) [file 13075_2019_1853_MOESM10_ESM.tif]
